# Association Study of *SPARCL1* Gene Polymorphisms in Ischemic Stroke

**DOI:** 10.3390/genes16091007

**Published:** 2025-08-26

**Authors:** Seong Shin Kwak, Ki Ook Lee, Chang Soo Ryu, Eun Ju Ko, Hyeon Woo Park, Jae Hyun Lee, Ok Joon Kim, Nam Keun Kim

**Affiliations:** 1 Department of Life Science, Graduate School, CHA University, 335 Pangyo-ro, Bundang-gu, Seongnam 13488, Republic of Korea; kkshin628@gmail.com (S.S.K.); regis2040@nate.com (C.S.R.); ejko05@naver.com (E.J.K.); aabb1114@naver.com (H.W.P.); athe7a@naver.com (J.H.L.); 2 Division of Life Sciences, College of Life Sciences, CHA University, 335 Pangyo-ro, Bundang-gu, Seongnam 13488, Republic of Korea; 3 Department of Neurology, CHA Bundang Medical Center, School of Medicine, CHA University, 59 Yatap-ro, Bundang-gu, Seongnam 13496, Republic of Korea; niceiatros@cha.ac.kr

**Keywords:** ischemic stroke, *SPARCL1*, hevin–calcyon binding, polymorphism

## Abstract

Background/Objectives: Stroke is a cerebrovascular disorder characterized by vessel occlusion or rupture, resulting in brain damage and subsequent physical impairment. Recent studies have implicated hevin–calcyon protein binding in the repair of brain injury. Secreted protein acidic and rich in cysteine–like 1 (*SPARCL1*) encodes hevin. This study investigated *SPARCL1* gene polymorphisms in ischemic stroke to identify potential biomarkers for brain injury treatment. Methods: we examined the associations of *SPARCL1* polymorphisms (rs1049544, rs1130643, rs7695558, rs1049539) with ischemic stroke. This case–control study involved 387 controls and 509 patients with ischemic stroke. Genotyping was performed via real-time polymerase chain reaction with the TaqMan™ SNP Genotyping Kit. Results: The rs1049544 polymorphism was significantly associated with ischemic stroke prevalence (GG vs. CC: adjusted odds ratio [AOR] = 0.642, *p* = 0.043; GG + GC vs. CC: AOR = 0.671, *p* = 0.045). Additionally, rs1049544 was significantly associated with large-artery disease prevalence (GG vs. CC: AOR = 0.489, *p* = 0.028; GG + GC vs. CC: AOR = 0.527, *p* = 0.033), and rs1130643 (TT vs. TC: AOR = 0.362, *p* = 0.039) was associated with cardioembolism prevalence in ischemic stroke subtype analysis. In haplotype analysis, G-G (rs1049544/rs7695558; odds ratio = 4.942, *p* = 0.001) and C-T (rs1049544/rs1049539; odds ratio = 0.776, *p* = 0.043) haplotypes were associated with ischemic stroke prevalence. Although some genotypes were not individually associated with ischemic stroke, the presence of the rs1049544 C allele appeared to enhance risk. Conclusions: These findings suggest that *SPARCL1* polymorphisms are associated with ischemic stroke and may be considered potential biomarkers for risk assessment.

## 1. Introduction

Stroke is a cerebrovascular disease that occurs when a blood vessel supplying the brain becomes blocked or ruptures, leading to brain damage and subsequent physical disabilities [1]. Stroke is the second-leading cause of death worldwide, and approximately 80% of all strokes are classified as ischemic [2]. Ischemic stroke, also termed cerebral infarction, results from necrosis of brain tissue due to arterial obstruction-mediated decreases in blood and oxygen supply [3]. Between 1990 and 2019, the absolute number of incident stroke cases increased by 70%, stroke prevalence rose by 85%, stroke-related deaths increased by 43%, and disability-adjusted life years attributed to stroke rose by 32% [4].

Ischemic stroke typically occurs when a blood clot or atherosclerotic plaque obstructs an artery supplying the brain [5]. In South Korea, stroke is the leading cause of death after cancer and is more prevalent than heart disease [6]. Numerous risk factors contribute to stroke, including hypertension, hyperlipidemia, alcohol abuse, brain tumors, myocardial infarction, coagulopathy, diabetes mellitus, and smoking [7]. Among these, hypertension [8,9], hyperlipidemia [10], diabetes mellitus [11,12], and smoking [13] are considered the most influential. Despite the identification of these risk factors, effective curative treatments for stroke remain limited. Thus, patients with stroke-related brain damage or traumatic brain injury often exclusively rely on rehabilitation therapy [14]. Recently, a novel mechanism involved in the neurodegenerative process after brain injury has been described. This mechanism centers on the regulation of the hevin–calcyon protein interactions to support synaptic repair. Hevin–calcyon binding plays a critical role in synaptic reconstruction during recovery [15]. Therefore, investigations of gene polymorphisms associated with ischemic stroke may help identify biomarkers related to hevin–calcyon protein binding, which could serve as targets for brain injury treatment.

**Figure 1:**
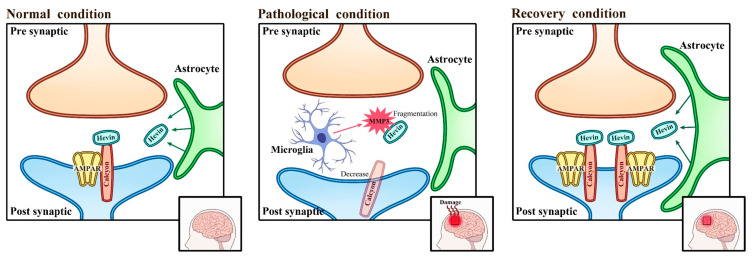
Mechanism of action of inflammatory activation enzymes in hevin–calcyon interaction. The process of repairing brain damage requires hevin-calcyon binding. *SPARCL1* interacts with synaptic adhesive proteins such as neurexin and neuroligin to regulate the formation of glutamate synapses in the developing brain. The brain icon represents neuronal connectivity.

SPARC-like protein 1 (*SPARCL1*), also known as hevin, is a synapse-inducing protein secreted by astrocytes [16,17]. The *SPARCL1* gene is located on chromosome 4 [18]. *SPARCL1* regulates the formation of glutamatergic synapses in the developing brain by interacting with synaptic adhesion proteins such as neurexin-1α and neuroligin-1 (NLG1) [15]. In addition to its synaptogenic function, hevin participates in calcium ion binding, collagen binding, and extracellular matrix binding; it is widely expressed in the brain, endometrium, and various other tissues [19]. Furthermore, *SPARCL1* has been implicated in anti-adhesive functions, inflammation regulation, and tumor suppression [20]. Hevin has been shown to promote glutamatergic synapse assembly by linking neurexin-1α and NLG1. Its synaptogenic function has been well characterized both in vivo and in vitro, demonstrating its critical role in bridging synaptic adhesion molecules. However, during early synaptic reconstruction, its activity is also evident during interactions with calcyon (rather than NLG1 or neurexin-1α). The extracellular domain of calcyon strongly binds to the C-terminal region of hevin, and this interaction may be essential for early synaptic recovery. Hevin utilizes this molecular interaction to interfere with calcyon-mediated α-amino-3-hydroxy-5-methyl-4-isoxazolepropionic acid (AMPA) receptor internalization in neurons. Thus, calcyon may play a more prominent role in synaptic adhesion by interacting with hevin rather than by regulating synaptic activity. As presented in Figure 1, enhanced hevin–calcyon binding accelerates neuronal connectivity in the brain, facilitating the restoration of damaged neural function. During this process, matrix metalloproteinase 3 (MMP3), induced by the inflammatory response, inhibits hevin–calcyon binding [15].

The clinical importance of 3′–untranslated region (UTR) miRNA binding site polymorphisms has received increasing attention in recent literature, given that such variants alter post-transcriptional regulation and influence susceptibility to diseases (e.g., cancer) [21]. In the context of cardiovascular and cerebrovascular diseases, polymorphisms in the 3′-UTR of the thymidylate synthase (TS) gene may serve as useful biomarkers for predicting and assessing the prognosis of ischemic stroke, silent brain infarction, and coronary artery disease [22,23]. Our previous study provided the first evidence to support 3′-UTR variants in TS as potential biomarkers for the prevalence of ischemic stroke, silent brain infarction, and coronary artery disease. Nitrogen permease regulator-like 3 (NPRL3) and N-methylpurine DNA glycosylase (MPG) genotypes and haplotypes may also offer clinically relevant biomarkers for the development, prevention, prognosis, and management of ischemic stroke [24].

Although *SPARCL1* has not been extensively studied in the context of ischemic stroke, its biological functions and expression profiles in other neurological diseases suggest a potential relevance. *SPARCL1* has been investigated in Alzheimer’s disease and multiple sclerosis, where its roles in neuroinflammation, astrocytic activation, and synaptic remodeling have been highlighted in experimental studies [25,26]. Given the central importance of astrocytic response and synaptic recovery in post-stroke repair, we hypothesized that genetic variants of *SPARCL1* may be associated with the prevalence and prognosis of ischemic stroke.

**Figure 2:**
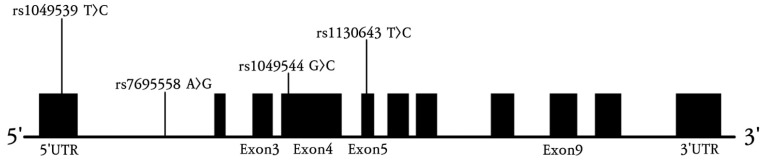
Overview of the four polymorphism locations within the *SPARCL1* gene employed in this study. The exon site is indicated by a black box. Exon 1 includes a 5′UTR, and Exon 11 includes a 3UTR.

Here, we conducted a genetic epidemiological study on single nucleotide polymorphisms (SNPs; *SPARCL1* rs1049539 T>C, rs7695558 A>G, rs1049544 G>C, rs1130643 T>C) to investigate associations of *SPARCL1* with the prevalence and prognosis of ischemic stroke in the Korean population. An overview of the genomic locations of these four polymorphisms within the *SPARCL1* gene is presented in Figure 2.

## 2. Materials and Methods

### 2.1. Study Population

This case–control study encompassed 387 control participants and 509 patients with ischemic stroke. All study participants were enrolled through the Department of Neurology at CHA Bundang Medical Center between 2000 and 2010. Ischemic stroke was defined as a stroke with evidence of cerebral infarction in clinically relevant brain regions based on magnetic resonance imaging (MRI) and electrocardiography findings.

WHO reports were cited in the Introduction to provide epidemiological context regarding stroke burden and classification; however, the WHO diagnostic criteria were not applied in patient selection because the standard of care in our institution prioritizes imaging-based confirmation over symptom duration alone.

According to clinical symptoms and neuroimaging data, two neurologists classified all pathological types of ischemic stroke into four subtypes using the Trial of ORG 10,172 in Acute Stroke Treatment (TOAST) criteria: (1) large-artery disease (LAD) comprised infarction lesions ≥ 15 mm in diameter on MRI, with significant (>50%) stenosis of a major cerebral artery or a branch cortical artery on cerebral angiography, accompanied by symptoms localized to the relevant arterial territory; (2) small-vessel disease (SVD) consisted of infarction lesions < 15 mm and ≥5 mm in diameter on MRI, accompanied by classic lacunar syndromes without evidence of cerebral cortical dysfunction or potential cardiac sources of embolism; (3) cardioembolism (CE) constituted arterial occlusion presumed to result from an embolus of cardiac origin, which was confirmed through cardiac evaluation; and (4) undetermined pathology was noted when the stroke cause could not be established due to insufficient data, the presence of multiple potential causes, or conflicting findings. The frequencies of stroke subtypes were as follows: LAD, 49% (*n* = 184); SVD, 36% (*n* = 136); and CE, 15% (*n* = 55), consistent with reported values for the Korean population. Single and multiple (≥2 lesions) SVDs were identified and distinguished using brain MRI.

The selection process for the 387 control participants was as follows. Eligible individuals were identified during routine medical examinations that included biochemical testing, electrocardiography, and brain MRI. These individuals visited the hospital not for treatment, but for general health screening purposes. Control participants (matched for sex and age) were selected from patients who had visited the hospital within the previous 5 years. Individuals with no recent history of stroke, cerebrovascular disease, or myocardial infarction were included in the study.

Hypertension was defined as having at least one systolic pressure > 140 mmHg and diastolic pressure > 90 mmHg and included patients who were currently taking hypertension medications. Diabetes mellitus was defined as a fasting blood glucose level > 126 mg/dL (7.0 mmol/L) and included patients taking antidiabetic medications. Smoking refers to individuals who currently smoke. Hyperlipidemia was defined as a history of high fasting serum total cholesterol level (≥240 mg/dL) or hyperlipidemia drug treatment.

### 2.2. Estimation of Biochemical Factors Concentration

We collected plasma samples to determine total homocysteine (tHcy) and folate levels within 48 h of stroke incidence. Whole blood was collected in tubes containing anticoagulants 12 h after the patient’s previous meal. The tubes were then centrifuged at 1000× *g* for 15 min to separate the plasma. Total tHcy concentration in plasma was measured using a fluorescence polarization immunoassay (FPIA) with an IMx system (Abott Laboratories, Chicago, IL, USA). Plasma folate concentrations were measured using a wireless assay kit (ACS 180; Bayer, Tarrytown, NY, USA). The levels of high-density lipoprotein cholesterol (HDL-C) were determined by an enzyme colorimetric method using a commercial reagent kit (TBA 200FR NEO, Toshiba Medical Systems, Tochigi, Japan).

### 2.3. Genotyping

Genomic DNA from stroke patients and controls was extracted from peripheral blood leukocytes using the G-DEX II Genomic DNA Extraction Kit (Intron Biotechnology, Seongnam, Korea). Genotyping of four *SPARCL1* polymorphisms (rs1049539 T>C, rs7695558 A>G, rs1049544 G>C, rs1130643 T>C) was conducted using the TaqMan™ SNP Genotype Analysis Kit (Thermo Fisher Scientific, Inc., Waltham, MA, USA) and the Rotor-Gene 6000 Real-Time PCR System (Qiagen Co., Ltd., Hilden, Germany). Genotyping validation and subtype analysis were performed by direct sequencing using polymerase chain reaction primers. All polymerase chain reaction primer sets used for sequencing are listed in Table S1.

### 2.4. Statistical Analysis

To compare clinical characteristics between patients with ischemic stroke and controls, the Chi-square test was applied to categorical variables; the t-test was used for continuous variables. Logistic regression analysis was performed to estimate associations between polymorphisms and ischemic stroke. Adjusted odds ratios (AORs) for *SPARCL1* polymorphisms were calculated with adjustments for age, sex, diabetes mellitus, hypertension, hyperlipidemia, and smoking status.

Odds ratios (ORs) and 95% confidence intervals (CIs) were calculated using Fisher’s exact test to determine associations of *SPARCL1* genotype frequencies with ischemic stroke. Additionally, ORs and 95% CIs were used to evaluate relationships between individual polymorphisms and allele combinations. The threshold for statistical significance was defined as *p* < 0.05. All polymorphisms were subjected to Hardy–Weinberg equilibrium analysis (*p* > 0.05). Continuous variables are presented as mean ± standard deviation (SD), and categorical variables as counts and percentages.

Data analysis was conducted using GraphPad Prism 4.0 (GraphPad Software Inc., San Diego, CA, USA), MedCalc version 22.017 (MedCalc Software, Mariakerke, Belgium), HAPSTAT 3.0 (University of North Carolina, Chapel Hill, NC, USA), and HaploView 4.1 (Broad Institute of MIT and Harvard, Boston, MA, USA). Multiple testing correction was performed using the Benjamini–Hochberg false discovery rate (FDR) method, implemented in R version 4.5.1 (R Foundation for Statistical Computing, Vienna, Austria) with RStudio (version 2025.05.1+513; Posit Software, Boston, MA, USA).

## 3. Results

### 3.1. Baseline Characteristics of Ischemic Stroke Patients and Control Groups

The demographic characteristics and clinical variables of patients with ischemic stroke and controls are summarized in Table 1. The proportion of male participants was 43.41% in the stroke group and 45.58% in the control group. The mean ages of stroke patients and controls were 62.81 ± 10.69 years and 64.00 ± 11.13 years, respectively. No significant differences in age or sex distribution were observed between the two groups. However, patients with ischemic stroke showed significantly higher rates of hypertension and diabetes mellitus, increases in homocysteine and fibrinogen, and decreases in folate and activated partial thromboplastin time (*p* < 0.05).

### 3.2. Genotype Frequencies of Four SPARCL1 Polymorphisms in Ischemic Stroke Patients and Controls

Four *SPARCL1* polymorphisms—rs1049539 T>C, rs7695558 A>G, rs1049544 G>C, and rs1130643 T>C—were examined. Table 2 presents the *SPARCL1* genotype frequencies in patients with ischemic stroke and control participants. AORs were calculated via logistic regression while controlling for age, sex, hypertension, diabetes mellitus, hyperlipidemia, and smoking. Genotype frequencies in both groups conformed to Hardy–Weinberg equilibrium expectations (*p* > 0.05). The *SPARCL1* rs1049544 G>C polymorphism showed a significant association with ischemic stroke prevalence (GG vs. CC: AOR = 0.642, 95% CI = 0.418–0.987, *p* = 0.043; GG + GC vs. CC: AOR = 0.672, 95% CI = 0.455–0.993, *p* = 0.046). However, rs1049539 T>C, rs7695558 A>G, and rs1130643 T>C polymorphisms were not significantly associated with ischemic stroke.

### 3.3. Genotype Frequencies of Four SPARCL1 Polymorphisms in Ischemic Stroke Subtypes and Controls

Subtype analyses identified associations between *SPARCL1* polymorphisms and specific ischemic stroke subtypes. LAD was significantly associated with rs1049544 G>C (GG vs. CC: AOR = 0.486, 95% CI = 0.257–0.920, *p* = 0.027; GG + GC vs. CC: AOR = 0.529, 95% CI = 0.294–0.953, *p* = 0.034). CE was significantly associated with rs1130643 T>C (TT vs. TC: AOR = 0.362, 95% CI = 0.138–0.949, *p* = 0.039) and rs1049539 T>C (TT vs. TC + CC: AOR = 1.883, 95% CI = 1.037–3.418, *p* = 0.038) (Table 3).

### 3.4. Analysis of SPARCL1 Haplotypes in Ischemic Stroke Patients and Controls

**Figure 3:**
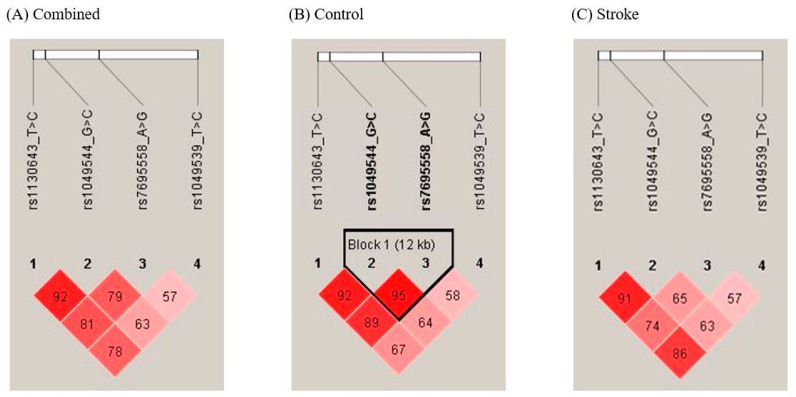
Linkage disequilibrium (LD) patterns of *SPARCL1* polymorphisms. The values in the squares denote LD between single markers. (**A**) LD patterns of *SPARCL1* polymorphisms in control and ischemic stroke patient subjects. No subjects exhibited an LD block. (**B**) LD patterns of *SPARCL1* polymorphisms in control subjects. Control subjects exhibited strong LD between *SPARCL1* rs1049544 G>C and rs7695558 A>G. (**C**) LD patterns of *SPARCL1* polymorphisms in ischemic stroke patient subjects. Ischemic stroke patients did not exhibit an LD block. Red squares indicate high values, and light squares indicate low values. Bold SNP numbers indicate markers involved in the strong LD block. LD, linkage disequilibrium; *SPARCL1*, secreted protein acidic and rich in cysteine-like 1.

Haplotype analysis was performed to compare patients with ischemic stroke and controls. Linkage disequilibrium among the four *SPARCL1* polymorphisms is presented in Figure 3. A strong linkage disequilibrium was observed between rs1049544 and rs7695558 in the control group. Among the four-marker haplotypes, T-A-C-C and T-G-G-T were more frequent in stroke patients than in controls, indicating increased susceptibility (T-A-C-C: OR = 4.434, 95% CI = 1.297–15.160; T-G-G-T: OR = 36.220, 95% CI = 2.195–597.800) (Table 4; full results in Table S2). In the three-marker haplotype analysis, the T-G-G (rs1049539/rs7695558/rs1049544), T-A-C and T-G-T (rs1049539/rs7695558/rs1130643), and G-G-T (rs7695558/rs1049544/rs1130643) haplotypes were more prevalent among stroke patients (T-G-G: OR = 7.655, 95% CI = 1.784–32.850; T-A-C: OR = 4.563, 95% CI = 1.572–13.250; T-G-T: OR = 3.042, 95% CI = 1.233–7.505; G-G-T: OR = 21.390, 95% CI = 2.902–157.700) (Table 4 and Table S2). In the two-marker haplotype analysis, the T-C (rs1049539/rs1049544), G-G (rs7695558/rs1049544), and C-C (rs1049544/rs1130643) haplotypes were associated with ischemic stroke prevalence (T-C: OR = 0.741, 95% CI = 0.570–0.962; G-G: OR = 6.338, 95% CI = 1.906–21.070; C-C: OR = 0.729, 95% CI = 0.536–0.991) (Table 4 and Table S2).

### 3.5. Combined Genotype Analysis of SPARCL1 Polymorphisms in Ischemic Stroke Patients and Controls

To confirm the effect of *SPARCL1* SNP genotypes on the risk of ischemic stroke, we performed a genotype combination analysis (Table 5, with full results in Tables S3 and S4).

For the three-site combinations, the *SPARCL1* genotype combinations of rs1049539/rs7695558/rs1049544 (TT/AG/CC: AOR = 0.117; TT/GG/CC: AOR = 0.276; TC/AG/CC: AOR = 0.425), rs1049539/rs1049544/rs1130643 (TT/CC/TC: AOR = 0.149), and rs7695558/rs1049544/rs1130643 (AG/CC/TC: AOR = 0.348) were associated with a reduced risk of ischemic stroke. Conversely, the rs1049539/rs7695558/rs1130643 (TT/AG/TT: AOR = 4.836) combination was associated with an increased risk (Table 5).

For two-site combinations, rs1049539/rs1049544 (TT/CC: AOR = 0.455), rs7695558/rs1049544 (AG/CC: AOR = 0.328), and rs1049544/rs1130643 (CC/TC: AOR = 0.421) were associated with a reduced risk of ischemic stroke. In contrast, the rs7695558/rs1049544 (AG/GG: AOR = 10.995) genotype combination was associated with an increased risk. Other genotype combinations were not significantly associated with ischemic stroke risk (Table 5).

### 3.6. Comparison of Clinical Variables Stratified by SPARCL1 Polymorphism Status in Ischemic Stroke Patients and Controls

To evaluate the potential associations between *SPARCL1* polymorphisms and clinical variables, ANOVA was performed after dividing participants into three groups: ischemic stroke patients and controls (Table S5), control group (Table S6), and ischemic stroke patients (Table S7). According to the ANOVA results for the ischemic stroke patients and controls, the *SPARCL1* rs1049539 T>C, rs7695558 A>G, and rs1130643 T>C were significantly associated with BMI (*p* = 0.048, 0.024, and 0.014, respectively), and rs7695558 A>G was also significantly associated with total cholesterol (*p* = 0.016) (Table S5). No significant associations were observed in the control group (Table S6).

In ischemic stroke patients, rs7695558 A>G and rs1130643 T>C were associated with BMI (*p* = 0.030 and 0.025), while rs7695558 A>G was also associated with total cholesterol (*p* = 0.023) and aPTT (*p* = 0.020). In addition, rs1049544 G>C showed a significant association with vitamin B12 levels (*p* = 0.039) (Table S7).

### 3.7. Stratified Analysis of SPARCL1 Genotype Frequencies by Clinical Parameters

Stratified analyses of the *SPARCL1* gene were conducted to investigate its association with ischemic stroke while accounting for various clinical factors, including age, sex, smoking, hypertension, diabetes mellitus, hyperlipidemia, HDL cholesterol, homocysteine, folate, vitamin B12, total cholesterol, triglycerides, platelet, PT, aPTT, fibrinogen, BUN, uric acid and D-dimer levels (Table S8).

The rs7695558 was significantly associated with a reduced risk of ischemic stroke in relation to HDL cholesterol and PT levels (Table S8). In addition, rs1049544 was also significantly associated with decreased stroke risk across several clinical factors, including sex, hypertension, diabetes mellitus, hyperlipidemia, smoking, triglycerides, vitamin B12, and platelets. Similarly, rs1130643 was significantly associated with decreased stroke risk in relation to smoking and total cholesterol level (Table S8). All stratified results are presented in Table S8.

### 3.8. Interaction Analysis Between SPARCL1 Polymorphisms and Clinical Factors Affecting Ischemic Stroke Prevalence

Interaction analysis was conducted to evaluate the potential synergistic effects between *SPARCL1* polymorphisms and clinical risk factors on ischemic stroke prevalence (Table S9).

rs1049539 showed significant interactions with hypertension, diabetes mellitus, hyperlipidemia, low HDL cholesterol levels, and low folate levels, indicating increased stroke risk in these subgroups. Similarly, rs7695558 demonstrated synergistic effects with hypertension, diabetes mellitus, smoking, and low folate levels, contributing to elevated risk. rs1049544 was also significantly associated with increased stroke risk when combined with hypertension, diabetes mellitus, hyperlipidemia, and low folate levels. In addition, rs1130643 exhibited significant interactions with hypertension, diabetes mellitus, smoking, and low folate levels, amplifying stroke risk in affected individuals. These findings suggest that specific *SPARCL1* polymorphisms may interact with vascular and metabolic risk factors to enhance susceptibility to ischemic stroke (Table S9).

## 4. Discussion

This study investigated the relationships of four *SPARCL1* polymorphisms with ischemic stroke to explore their potential relevance as genetic risk factors. Among SNPs with putative functional significance, *SPARCL1* rs1049539 T>C, rs7695558 A>G, rs1049544 G>C, and rs1130643 T>C were selected for analysis. Analysis of clinical characteristics revealed significant differences between patients with ischemic stroke and controls in terms of hypertension, diabetes mellitus, homocysteine, folate, activated partial thromboplastin time, and fibrinogen. The *SPARCL1* rs1049544 G>C genotype showed a significant association with ischemic stroke and was specifically linked to the LAD subtype. Although rs1049539 T>C and rs1130643 T>C were not significantly associated with stroke in overall analyses, subtype analysis revealed significant associations with the CE subtype. Further analysis of interactions between *SPARCL1* polymorphisms and risk factors demonstrated a synergistic effect on stroke risk under the recessive model for rs1049544 (Table S9). Haplotype analysis indicated that the *SPARCL1* rs1049539 T, rs1049539 C, rs7695558 G, rs1049544 G, rs1049544 C, and rs1130643 C alleles were associated with increased ischemic stroke incidence. Notably, rs1130643 C and rs1049539 C alleles also were associated with reduced prevalence in several haplotype combinations. This discrepancy may be explained by haplotype-specific interactions or linkage disequilibrium between adjacent loci. As such, this potential limitation should be considered when interpreting the results, and further studies with larger and more diverse cohorts are warranted. Given these observations, the potential biological mechanisms underlying the observed associations warrant consideration. *SPARCL1* is involved in extracellular matrix interactions and neurovascular remodeling, processes essential for maintaining the integrity of the blood–brain barrier and promoting neural recovery after injury. Variations in *SPARCL1* may alter vascular stability and synaptic plasticity, thereby influencing susceptibility to ischemic damage. The genomic positions of the four studied SNPs are presented in Figure 2. These findings provide evidence of potential relationships between *SPARCL1* polymorphisms and ischemic stroke.

Previous studies of *SPARCL1* have identified associations with conditions such as Alzheimer’s disease, colon cancer, pulmonary hypertension, and pancreatic cancer [25,27,28]. SPARC has demonstrated high expression in the brains of patients with Alzheimer’s disease, where it binds to Aβ protein deposits, actively contributing to neuroinflammation and subsequent tissue repair [25]. Additionally, hevin expression is reportedly downregulated under pathological conditions. Recent evidence indicates that *SPARCL1*, a synapse-inducing protein secreted by astrocytes, regulates the formation of glutamatergic synapses in the developing brain through interactions with synaptic adhesion proteins (e.g., neurexin-1α and NLG1) [29]. Furthermore, the neuron-specific vesicular protein calcyon has been identified as a novel interaction partner of *SPARCL1*; this interaction plays a pivotal role in synaptic reconstruction after injury in the mature brain [15]. These synapse-related functions of *SPARCL1* may have implications for stroke risk, as efficient synaptic reconstruction and maintenance of neurovascular integrity are critical in mitigating the effects of ischemic injury. Disruption of these processes, potentially influenced by genetic variation in *SPARCL1*, could impair post-ischemic recovery and increase susceptibility to stroke. The *SPARCL1*–calcyon interaction may be particularly relevant in the context of ischemic stroke, given that early synaptic recovery is critical for functional restoration.

This study matched cases and controls for age and sex; however, other conventional stroke risk factors such as smoking, hypertension, diabetes mellitus, and hyperlipidemia were more prevalent in the ischemic stroke group, reflecting the real-world characteristics of stroke patients. Although achieving identical distributions of these risk factors between groups is practically unfeasible in this disease context, we addressed potential confounding by performing multivariable logistic regression, adjusting for these variables. Importantly, the associations between *SPARCL1* polymorphisms and ischemic stroke remained statistically significant after adjustment.

In this study, we found that the *SPARCL1* rs1049539 T>C, rs1049544 G>C, and rs1130643 T>C polymorphisms were significantly associated with ischemic stroke prevalence, whereas the rs7695558 A>G polymorphism showed no significant association. Notably, genotypes not initially associated with stroke risk demonstrated increased risk when the rs1049544 C allele was present. Based on these findings, we propose that *SPARCL1* polymorphisms influence the risk of ischemic stroke and may be useful as genetic markers for risk assessment. These variants could also represent candidates for early risk prediction, supporting targeted prevention strategies, consistent with prior studies evaluating the clinical relevance of functional gene polymorphisms, such as those in the TS gene [22,23]. Similarly, functional polymorphisms in the *MUC4* gene have been significantly associated with idiopathic recurrent pregnancy loss, reinforcing the role of missense variants in disease susceptibility and their potential utility as predictive biomarkers [30].

## 5. Conclusions

Although this study confirmed a genetic association between ischemic stroke and *SPARCL1*, some limitations should be acknowledged. First, the underlying mechanism by which *SPARCL1* polymorphisms influence stroke development remains unclear, although our findings and prior evidence suggest that *SPARCL1* may contribute to stroke susceptibility through roles in extracellular matrix interactions, neurovascular remodeling, and maintenance of blood–brain barrier integrity. Second, the absence of data concerning additional environmental risk factors among stroke patients warrants further investigation. Third, the study population was limited to individuals of Korean ethnicity. Notably, the sample size included fewer than 1000 individuals, but the use of a relatively homogeneous population supports the reliability of the findings. However, validation in diverse racial and ethnic groups is necessary. If future studies establish a definitive causal role for the *SPARCL1* pathway in ischemic stroke, targeted prevention strategies may become feasible. Thus, further epidemiological research involving heterogeneous populations is essential to deepen understanding of the relationship between *SPARCL1* polymorphisms and ischemic stroke prevalence. Readers should interpret these findings with the understanding that they are derived from a Korean population-based study.

## Figures and Tables

**Table 1:** Baseline characteristics of ischemic stroke patients and control groups.

Characteristic	Controls (*n* = 387)	Stroke Patients (*n* = 509)	*p* ^a^
Male (%)	168 (43.41)	232 (45.58)	0.734
Age (years, mean ± SD)	62.81 ± 10.69	64.00 ± 11.13	0.110
Smoking (%)	129 (33.33)	203 (39.88)	0.193
Hypertension (%)	162 (41.86)	327 (64.24)	**0.0003**
Diabetes mellitus (%)	55 (14.21)	141 (27.70)	**0.0001**
Hyperlipidemia (%)	88 (22.74)	150 (29.47)	0.098
BMI (kg/m^2^, mean ± SD)	24.25 ± 3.21	24.21 ± 3.82	0.877
HDL-C (mg/dL, mean ± SD)	46.48 ± 13.86	44.80 ± 15.65	0.222
Homocysteine (μmol/L, mean ± SD)	10.05 ± 4.09	11.21 ± 6.60	**0.003**
Folate (nmol/L, mean ± SD)	8.93 ± 8.11	6.83 ± 4.94	**<0.0001**
Vitamin B12 (pg/mL, mean ± SD)	687.18 ± 277.40	690.66 ± 322.64	0.867
Total cholesterol (mg/dL, mean ± SD)	193.04 ± 37.78	189.89 ± 40.91	0.244
Triglycerides (mg/dL, mean ±SD)	145.78 ± 87.56	151.09 ± 111.27	0.445
PLT (103/μL, mean ± SD)	244.12 ± 65.63	244.76 ± 87.56	0.905
PT (s, mean ± SD)	11.78 ± 0.78	11.93 ± 3.31	0.469
aPTT (s, mean ± SD)	32.32 ± 9.09	30.56 ± 4.44	**0.0003**
Fibrinogen (mg/dL, mean ± SD)	396.92 ± 121.97	427.92 ± 130.48	**0.014**
Antithrombin (%, mean ± SD)	94.48 ± 44.58	92.57 ± 16.77	0.446
BUN (mg/dL, mean ± SD)	16.02 ± 5.01	16.19 ± 6.25	0.667
Uric acid (mg/dL, mean ± SD)	4.72 ± 1.46	4.64 ± 1.53	0.466

SD, standard deviation; BMI, body mass index; HDL-C, high-density lipoprotein cholesterol; PLT, platelet; PT, prothrombin time; aPTT, activated partial thromboplastin time; BUN, blood urea nitrogen. ^a^* p*-values were calculated via a two-sided Student’s t-test for continuous variables and a Chi-square test for categorical variables. Bold values indicate statistical significance (*p *< 0.05).

**Table 2:** Genotype frequencies of four *SPARCL1* polymorphisms in ischemic stroke patients and control groups.

Genotypes	Controls (*n* = 387)	Stroke Patients (*n* = 509)	COR (95% CI)	*p*	*FDR-* *P*	AOR (95% CI) ^a^	*p*	*FDR-* *P*
*SPARCL1* rs1049539 T>C								
TT	194 (50.13)	247 (48.53)	1.000 (reference)			1.000 (reference)		
TC	162 (41.86)	227 (44.60)	1.100 (0.835–1.550)	0.496	0.661	1.069 (0.801–1.425)	0.652	0.652
CC	31 (8.01)	35 (6.88)	0.887 (0.528–1.490)	0.650	0.650	0.923 (0.535–1.593)	0.774	0.774
Dominant (TT vs. TC + CC)			1.066 (0.819–1.390)	0.635	0.635	1.045 (0.792–1.378)	0.756	0.756
Recessive (TT + TC vs. CC)			0.848 (0.513–1.402)	0.521	0.679	0.893 (0.526–1.515)	0.675	0.819
HWE *P*	0.727	0.074						
*SPARCL1* rs7695558 A>G								
AA	289 (74.68)	401 (78.78)	1.000 (reference)			1.000 (reference)		
AG	88 (22.74)	97 (19.06)	0.794 (0.574–1.100)	0.166	0.332	0.816 (0.579–1.149)	0.244	0.489
GG	10 (2.58)	11 (2.16)	0.793 (0.332–1.892)	0.601	0.601	0.874 (0.354–2.156)	0.770	0.774
Dominant (AA vs. AG + GG)			0.794 (0.581–1.086)	0.149	0.298	0.822 (0.592–1.141)	0.240	0.343
Recessive (AA + AG vs. GG)			0.833 (0.350–1.981)	0.679	0.679	0.900 (0.366–2.216)	0.819	0.819
HWE *P*	0.297	0.082						
*SPARCL1* rs1049544 G>C								
GG	134 (34.63)	193 (37.92)	1.000 (reference)			1.000 (reference)		
GC	184 (47.55)	254 (49.90)	0.958 (0.717–1.282)	0.775	0.775	0.922 (0.680–1.250)	0.600	0.652
CC	69 (17.83)	62 (12.18)	0.624 (0.415–0.938)	**0.023**	**0.023**	0.642 (0.418–0.986)	**0.043**	0.171
Dominant (GG vs. GC + CC)			0.867 (0.658–1.142)	0.311	0.415	0.846 (0.634–1.130)	0.257	0.343
Recessive (GG + GC vs. CC)			0.639 (0.441–0.927)	**0.018**	0.072	0.672 (0.455–0.993)	**0.046**	0.184
HWE *P*	0.764	0.120						
*SPARCL1* rs1130643 T>C								
TT	296 (76.49)	413 (81.14)	1.000 (reference)			1.000 (reference)		
TC	83 (21.45)	88 (17.29)	0.760 (0.544–1.062)	0.108	0.332	0.786 (0.553–1.116)	0.178	0.489
CC	8 (2.07)	8 (1.57)	0.717 (0.266–1.931)	0.510	0.510	0.790 (0.282–2.214)	0.654	0.774
Dominant (TT vs. TC + CC)			0.756 (0.547–1.045)	0.090	0.298	0.786 (0.560–1.103)	0.163	0.343
Recessive (TT + TC vs. CC)			0.757 (0.281–2.034)	0.580	0.679	0.834 (0.298–2.336)	0.729	0.819
HWE *P*	0.447	0.194						

COR, crude odds ratio; AOR, adjusted odds ratio; HWE, Hardy–Weinberg equilibrium; 95% CI, 95% confidence interval; N/A, not applicable; *SPARCL1*, secreted protein acidic and rich in cysteine-like 1. ^a^ Adjusted by age, sex, hypertension, diabetes mellitus, hyperlipidemia, and smoking. *P* indicates *p*-values for Hardy–Weinberg equilibrium (HWE). Bold values indicate statistical significance (*p *< 0.05 or FDR-*p* < 0.05).

**Table 3:** Genotype frequencies of four *SPARCL1* polymorphisms between ischemic stroke subtype and controls.

Genotypes	Controls (*n* = 387)	LAD (*n* = 184)	AOR (95% CI) ^a^	*p*	*FDR-P*	SVD (*n* = 136)	AOR (95% CI) ^a^	*p*	*FDR-P*	CE (*n* = 55)	AOR (95% CI) ^a^	*p*	*FDR-P*
*SPARCL1* rs1049539 T>C													
TT	194 (50.13)	84 (45.65)	1.000 (reference)			76 (55.88)	1.000 (reference)			20 (36.36)	1.000 (reference)		
TC	162 (41.86)	86 (46.74)	1.153 (0.785–1.694)	0.467	0.846	56 (41.18)	0.889 (0.585–1.351)	0.582	0.846	29 (52.73)	1.849 (0.993–3.442)	0.053	0.427
CC	31 (8.01)	14 (7.61)	1.097 (0.534–2.255)	0.802	0.913	4 (2.94)	0.355 (0.117–1.080)	0.068	0.427	6 (10.91)	2.449 (0.861–6.971)	0.093	0.498
Dominant (TT vs. TC + CC)			1.146 (0.791–1.660)	0.472	0.846		0.804 (0.534–1.209)	0.295	0.846		1.883 (1.037–3.418)	**0.038**	0.427
Recessive (TT + TC vs. CC)			1.049 (0.523–2.105)	0.893	0.953		0.368 (0.125–1.082)	0.069	0.427		1.720 (0.665–4.452)	0.264	0.846
*SPARCL1* rs7695558 A>G													
AA	289 (74.68)	146 (79.35)	1.000 (reference)			101 (74.26)	1.000 (reference)			44 (80.00)	1.000 (reference)		
AG	88 (22.74)	36 (19.57)	0.911 (0.575–1.443)	0.690	0.913	30 (22.06)	0.943 (0.574–1.551)	0.818	0.913	9 (16.36)	0.667 (0.309–1.441)	0.303	0.846
GG	10 (2.58)	2 (1.09)	0.427 (0.087–2.091)	0.293	0.846	5 (3.68)	1.669 (0.534–5.217)	0.379	0.846	2 (3.64)	1.263 (0.255–6.255)	0.775	0.913
Dominant (AA vs. AG + GG)			0.859 (0.550–1.343)	0.505	0.846		1.012 (0.633–1.618)	0.962	0.982		0.727 (0.356–1.486)	0.382	0.846
Recessive (AA + AG vs. GG)			0.431 (0.088–2.106)	0.299	0.846		1.666 (0.535–5.191)	0.379	0.846		1.386 (0.282–6.822)	0.688	0.913
*SPARCL1* rs1049544 G>C													
GG	134 (34.63)	70 (38.04)	1.000 (reference)			50 (36.76)	1.000 (reference)			18 (32.73)	1.000 (reference)		
GC	185 (47.80)	97 (52.72)	0.928 (0.620–1.389)	0.716	0.913	65 (47.79)	0.995 (0.637–1.555)	0.982	0.982	25 (45.45)	1.016 (0.528–1.957)	0.962	0.982
CC	68 (17.57)	17 (9.24)	0.486 (0.257–0.920)	**0.027**	0.427	21 (15.44)	0.800 (0.426–1.502)	0.488	0.846	12 (21.82)	1.323 (0.587–2.978)	0.500	0.846
Dominant (GG vs. GC + CC)			0.834 (0.568–1.224)	0.353	0.846		0.939 (0.614–1.437)	0.773	0.913		1.101 (0.598–2.026)	0.758	0.913
Recessive (GG + GC vs. CC)			0.529 (0.294–0.953)	**0.034**	0.427		0.848 (0.487–1.476)	0.560	0.846		1.287 (0.636–2.604)	0.483	0.846
*SPARCL1* rs1130643 T>C													
TT	296 (76.49)	155 (84.24)	1.000 (reference)			102 (75.00)	1.000 (reference)			48 (87.27)	1.000 (reference)		
TC	83 (21.45)	27 (14.67)	0.688 (0.418–1.133)	0.142	0.620	32 (23.53)	1.150 (0.708–1.869)	0.572	0.846	5 (9.09)	0.362 (0.138–0.949)	**0.039**	0.427
CC	8 (2.07)	2 (1.09)	0.549 (0.106–2.850)	0.475	0.846	2 (1.47)	0.858 (0.170–4.335)	0.853	0.930	2 (3.64)	1.588 (0.307–8.220)	0.582	0.846
Dominant (TT vs. TC + CC)			0.678 (0.418–1.098)	0.114	0.548		1.127 (0.702–1.809)	0.621	0.876		0.461 (0.198–1.069)	0.071	0.427
Recessive (TT + TC vs. CC)			0.597 (0.116–3.081)	0.538	0.846		0.814 (0.160–4.134)	0.804	0.913		1.918 (0.374–9.846)	0.435	0.846

AOR, adjusted odds ratio; 95% CI, 95% confidence interval; LAD, large-artery disease; SVD, small-vessel disease; CE, cardioembolism; N/A, not applicable; *SPARCL1*, secreted protein acidic and rich in cysteine-like 1. ^a^ Adjusted for age, sex, hypertension, diabetes mellitus, hyperlipidemia, and smoking. Bold values indicate statistical significance (*p *< 0.05).

**Table 4:** Analysis of *SPARCL1* haplotypes in ischemic stroke patients and controls.

Haplotype	Stroke Controls (*2n* = 774)	Stroke Patients (*2n* = 1018)	OR (95% CI)	*p* ^a^	*FDR-P*
*SPARCL1* rs1049539 T>C/rs7695558 A>G/rs1049544 G>C/rs1130643 T>C					
T-A-G-T	402 (51.94)	544 (53.44)	1.000 (reference)		
T-A-C-C	3 (0.39)	18 (1.77)	4.434 (1.297–15.160)	**0.012**	0.056
T-G-G-T	0 (0)	24 (2.36)	36.220 (2.195–597.800)	**<0.0001**	**0.001**
*SPARCL1* rs1049539 T>C/rs7695558 A>G/rs1049544 G>C					
T-A-G	401 (51.81)	550 (54.03)	1.000 (reference)		
T-G-G	2 (0.26)	21 (2.06)	7.655 (1.784–32.850)	**0.001**	**0.007**
*SPARCL1* rs1049539 T>C/rs7695558 A>G/rs1130643 T>C					
T-A-T	454 (58.66)	597 (58.64)	1.000 (reference)		
T-A-C	4 (0.52)	24 (2.36)	4.563 (1.572–13.250)	**0.002**	**0.014**
T-G-T	6 (0.78)	24 (2.36)	3.042 (1.233–7.505)	**0.011**	**0.035**
*SPARCL1* rs7695558 A>G/rs1049544 G>C/rs1130643 T>C					
A-G-T	447 (57.75)	606 (59.53)	1.000 (reference)		
G-G-T	1 (0.13)	29 (2.85)	21.390 (2.902–157.700)	**<0.0001**	**0.001**
*SPARCL1* rs1049539 T>C/rs1049544 G>C					
T-G	406 (52.45)	571 (56.09)	1.000 (reference)		
T-C	144 (18.60)	150 (14.73)	0.741 (0.570–0.962)	**0.024**	0.072
*SPARCL1* rs7695558 A>G/rs1049544 G>C					
A-G	449 (58.01)	614 (60.31)	1.000 (reference)		
G-G	3 (0.39)	26 (2.55)	6.338 (1.906–21.070)	**0.0004**	**0.001**
*SPARCL1* rs1049544 G>C/rs1130643 T>C					
G-T	448 (57.88)	634 (62.28)	1.000 (reference)		
C-C	95 (12.27)	98 (9.63)	0.729 (0.536–0.991)	**0.043**	0.129

OR, odds ratio; 95% CI, 95% confidence interval; *SPARCL1*, secreted protein acidic and rich in cysteine-like 1. ^a^ Bold values indicate statistical significance (*p* < 0.05 or FDR-*p* < 0.05).

**Table 5:** Combined genotype analysis for *SPARCL1* polymorphisms in ischemic stroke patients and controls.

Genotype	Stroke Controls (*n* = 387)	Stroke Patients (*n* = 509)	AOR (95% CI) ^a^	*p*	*FDR-P*
*SPARCL1* rs1049539 T>C/rs7695558 A>G/rs1049544 G>C					
TT/AA/GG	106 (27.39)	143 (28.09)	1.000 (reference)		
TT/AG/CC	8 (2.07)	2 (0.39)	0.117 (0.021–0.634)	**0.013**	0.208
TT/GG/CC	9 (2.33)	4 (0.79)	0.276 (0.077–0.993)	**0.049**	0.261
TC/AG/CC	19 (4.91)	12 (2.36)	0.425 (0.187–0.968)	**0.042**	0.261
*SPARCL1* rs1049539 T>C/rs7695558 A>G/rs1130643 T>C					
TT/AA/TT	133 (34.37)	168 (33.01)	1.000 (reference)		
TT/AG/TT	2 (0.52)	11 (2.16)	4.836 (1.009–23.173)	**0.049**	0.366
*SPARCL1* rs1049539 T>C/rs1049544 G>C/rs1130643 T>C					
TT/GG/TT	105 (27.13)	150 (29.47)	1.000 (reference)		
TT/CC/TC	10 (2.58)	3 (0.59)	0.149 (0.036–0.610)	**0.008**	0.120
*SPARCL1* rs7695558 A>G/rs1049544 G>C/rs1130643 T>C					
AA/GG/TT	131 (33.85)	176 (34.58)	1.000 (reference)		
AG/CC/TC	25 (6.46)	14 (2.75)	0.348 (0.161–0.749)	**0.007**	0.091
*SPARCL1* rs1049539 T>C/rs1049544 G>C					
TT/GG	107 (27.65)	152 (29.86)	1.000 (reference)		
TT/CC	19 (4.91)	14 (2.75)	0.455 (0.211–0.980)	**0.044**	0.352
*SPARCL1* rs7695558 A>G/rs1049544 G>C					
AA/GG	133 (34.37)	179 (35.17)	1.000 (reference)		
AG/GG	1 (0.26)	13 (2.55)	10.995 (1.370–88.261)	**0.024**	0.084
AG/CC	28 (7.24)	15 (2.95)	0.328 (0.158–0.683)	**0.003**	**0.021**
*SPARCL1* rs1049544 G>C/rs1130643 T>C					
GG/TT	131 (33.85)	190 (37.33)	1.000 (reference)		
CC/TC	28 (7.24)	19 (3.73)	0.421 (0.214–0.826)	**0.012**	0.072

AOR, adjusted odds ratio; 95% CI, 95% confidence interval; *SPARCL1*, secreted protein acidic and rich in cysteine-like 1. ^a^ Adjusted for age, sex, hypertension, diabetes mellitus, hyperlipidemia, and smoking. Bold values indicate statistical significance (*p *< 0.05 or FDR-*p* < 0.05).

## Data Availability

All supporting data used in the study are available from the corresponding authors upon request.

## Supplementary Materials

The following supporting information can be downloaded at: https://www.mdpi.com/article/10.3390/genes16091007/s1, Table S1: Information on *SPARCL1* primer set for sequencing; Table S2: Analysis of *SPARCL1* haplotypes in ischemic stroke patients and controls; Table S3: Combined genotype analysis for *SPARCL1* polymorphisms in ischemic stroke patients and controls (Set 1); Table S4: Combined genotype analysis for *SPARCL1* polymorphisms in ischemic stroke patients and controls (Set 2); Table S5: Differences between clinical variables in ischemic stroke patients and controls stratified by *SPARCL1* polymorphism status via ANOVA; Table S6: Differences between clinical variables in controls stratified by *SPARCL1* polymorphism status via ANOVA; Table S7: Differences between clinical variables in ischemic stroke patients stratified by *SPARCL1* polymorphism status via ANOVA; Table S8: Stratified analysis using clinical parameters with frequency of *SPARCL1* genotypes; Table S9: The synergistic effect analysis of the interplay between clinical factors and *SPARCL1* polymorphisms in ischemic stroke prevalence.
