# Supplementary material for: Association Study of SPARCL1 Gene Polymorphisms in Ischemic Stroke
Source: Genes (Basel). 2025 Aug 26;16(9):1007. doi: 10.3390/genes16091007 (PMC12469447; doi:10.3390/genes16091007)
Supplement: Supplementary file 1 [file genes-16-01007-s001.zip › genes-3801460-supplementary.pdf]

Table S1. Information of *SPARCL1* primer set for sequencing

Gene	SNP	Region	Method	Primer sequence		Product size	Annealing temperature
SPARCL1	rs1049539	5 prime UTR	TaqMan	Forward	5'-ATT GAA GTT GAA TCC CCT CTG-3'	223 bp	53 °C
SPARCL1				Reverse	5'-CGA TGA CAG TCT GAA ATA CTC-3'		
SPARCL1	rs7695558	intron	TaqMan	Forward	5'-AAC TCA ACA TCA TTG GTT CTC AGG-3'	730 bp	57 °C
SPARCL1				Reverse	5'-GAT CAT GTG AGA TCA TGC GGA CAG-3'		
SPARCL1	rs1049544	missense	TaqMan	Forward	5'-GGA ACT AAC ACC AGG AGC CA-3'	227bp	55 °C
SPARCL1				Reverse	5'-CAG TAC TAA AGT CAA AAG AGG-3'		
SPARCL1	rs1130643	missense	TaqMan	Forward	5'-CAA GCC CAA AGT GGC AGA TT-3'	227 bp	56 °C
SPARCL1				Reverse	5'-GTG AAA GTA TGC ACA GTG GAA AT-3'		

Table S2. Analysis of *SPARCL1* haplotypes in ischemic stroke patients and controls

Haplotype	Stroke Controls (2*n* = 774)	Stroke Patients (2*n* = 1018)	OR (95% CI)	*P* [a]	*FDR-P*
SPARCL1 rs1049539 T>C/rs7695558 A>G/rs1049544 G>C/rs1130643 T>C					
T-A-G-T	402 (51.94)	544 (53.44)	1.000 (reference)		
T-A-G-C	2 (0.26)	5 (0.49)	1.847 (0.357 - 9.574)	0.705	0.823
T-A-C-T	52 (6.72)	54 (5.30)	0.767 (0.513 - 1.147)	0.196	0.457
T-A-C-C	3 (0.39)	18 (1.77)	4.434 (1.297 - 15.160)	**0.012**	0.056
T-G-G-T	0 (0)	24 (2.36)	36.220 (2.195 - 597.800)	**<0.0001**	**0.001**
T-G-G-C	1 (0.13)	0 (0.00)	0.246 (0.010 - 6.069)	0.426	0.669
T-G-C-T	7 (0.9)	0 (0.00)	0.049 (0.003 - 0.866)	**0.003**	**0.021**
T-G-C-C	83 (10.72)	76 (7.47)	0.677 (0.483 - 0.948)	**0.023**	0.081
C-A-G-T	44 (5.68)	62 (6.09)	1.041 (0.693 - 1.565)	0.846	0.911
C-A-G-C	1 (0.13)	0 (0.00)	0.246 (0.010 - 6.069)	0.426	0.669
C-A-C-T	159 (20.54)	216 (21.22)	1.004 (0.788 - 1.279)	0.975	0.975
C-A-C-C	4 (0.52)	0 (0.00)	0.082 (0.004 - 1.531)	**0.033**	0.092
C-G-G-T	2 (0.26)	4 (0.39)	1.478 (0.269 - 8.112)	0.651	0.823
C-G-G-C	0 (0)	0 (0.00)	N/A	N/A	N/A
C-G-C-T	10 (1.29)	10 (0.98)	0.739 (0.305 - 1.793)	0.502	0.703
C-G-C-C	5 (0.65)	4 (0.39)	0.591 (0.158 - 2.216)	0.430	0.669
SPARCL1 rs1049539 T>C/rs7695558 A>G/rs1049544 G>C					
T-A-G	401 (51.81)	550 (54.03)	1.000 (reference)		
T-A-C	54 (6.98)	71 (6.97)	0.959 (0.658 - 1.397)	0.826	0.989
T-G-G	2 (0.26)	21 (2.06)	7.655 (1.784 - 32.850)	**0.001**	**0.007**
T-G-C	92 (11.89)	79 (7.76)	0.626 (0.451 - 0.868)	**0.005**	**0.018**
C-A-G	46 (5.94)	64 (6.29)	1.014 (0.680 - 1.514)	0.944	0.989
C-A-C	164 (21.19)	214 (21.02)	0.951 (0.748 - 1.210)	0.685	0.989
C-G-G	2 (0.26)	4 (0.39)	1.458 (0.266 - 8.003)	0.662	0.989
C-G-C	11 (1.42)	15 (1.47)	0.994 (0.452 - 2.188)	0.989	0.989
SPARCL1 rs1049539 T>C/rs7695558 A>G/rs1130643 T>C					
T-A-T	454 (58.66)	597 (58.64)	1.000 (reference)		
T-A-C	4 (0.52)	24 (2.36)	4.563 (1.572 - 13.250)	**0.002**	**0.014**
T-G-T	6 (0.78)	24 (2.36)	3.042 (1.233 - 7.505)	**0.011**	**0.035**
T-G-C	85 (10.98)	76 (7.47)	0.680 (0.488 - 0.948)	**0.023**	**0.040**
C-A-T	202 (26.1)	278 (27.31)	1.047 (0.841 - 1.302)	0.683	0.855
C-A-C	5 (0.65)	0 (0.00)	0.069 (0.004 - 1.255)	**0.015**	**0.035**
C-G-T	12 (1.55)	15 (1.47)	0.951 (0.441 - 2.051)	0.897	0.897
C-G-C	4 (0.52)	4 (0.39)	0.761 (0.189 - 3.058)	0.733	0.855
SPARCL1 rs1049539 T>C/rs1049544 G>C/rs1130643 T>C					
T-G-T	403 (52.07)	568 (55.80)	1.000 (reference)		
T-G-C	3 (0.39)	5 (0.49)	1.183 (0.281 - 4.978)	0.819	0.845
T-C-T	58 (7.49)	54 (5.30)	0.661 (0.446 - 0.978)	**0.037**	0.179
T-C-C	85 (10.98)	94 (9.23)	0.785 (0.570 - 1.081)	0.137	0.320
C-G-T	45 (5.81)	66 (6.48)	1.041 (0.698 - 1.552)	0.845	0.845
C-G-C	1 (0.13)	1 (0.10)	0.710 (0.044 - 11.380)	0.808	0.845
C-C-T	169 (21.83)	226 (22.20)	0.949 (0.749 - 1.202)	0.663	0.845
C-C-C	9 (1.16)	4 (0.39)	0.315 (0.096 - 1.031)	0.051	0.179
SPARCL1 rs7695558 A>G/rs104954 G>C/rs1130643 T>C					
A-G-T	447 (57.75)	606 (59.53)	1.000 (reference)		
A-G-C	3 (0.39)	6 (0.59)	1.475 (0.367 - 5.933)	0.741	0.741
A-C-T	210 (27.13)	269 (26.42)	0.945 (0.760 - 1.175)	0.610	0.711
A-C-C	7 (0.9)	18 (1.77)	1.897 (0.785 - 4.581)	0.148	0.253
G-G-T	1 (0.13)	29 (2.85)	21.390 (2.902 - 157.700)	**<0.0001**	**0.001**
G-G-C	2 (0.26)	0 (0.00)	0.148 (0.007 - 3.084)	0.181	0.253
G-C-T	17 (2.2)	10 (0.98)	0.434 (0.197 - 0.957)	**0.034**	**0.079**
G-C-C	88 (11.37)	80 (7.86)	0.671 (0.484 - 0.930)	**0.016**	0.056
SPARCL1 rs1049539 T>C/rs7695558 A>G					
T-A	455 (58.79)	617 (60.61)	1.000 (reference)		
T-G	95 (12.27)	104 (10.22)	0.807 (0.596 - 1.094)	0.166	0.498
C-A	211 (27.26)	282 (27.70)	0.986 (0.794 - 1.223)	0.895	0.895
C-G	13 (1.68)	15 (1.47)	0.851 (0.401 - 1.806)	0.674	0.895
SPARCL1 rs1049539 T>C/rs1049544 G>C					
T-G	406 (52.45)	571 (56.09)	1.000 (reference)		
T-C	144 (18.60)	150 (14.73)	0.741 (0.570 - 0.962)	**0.024**	0.072
C-G	46 (5.94)	69 (6.78)	1.067 (0.719 - 1.582)	0.749	0.749
C-C	178 (23)	228 (22.40)	0.911 (0.721 - 1.151)	0.433	0.650
SPARCL1 rs1049539 T>C/rs1130643 T>C					

T-T	460 (59.43)	621 (61.00)	1.000 (reference)		
T-C	90 (11.63)	100 (9.82)	0.823 (0.604 - 1.121)	0.217	0.326
C-T	215 (27.78)	293 (28.78)	1.009 (0.816 - 1.250)	0.931	0.931
C-C	9 (1.16)	4 (0.39)	0.329 (0.101 - 1.076)	0.087	0.261
SPARCL1 rs7695558 A>G/rs1049544 G>C					
A-G	449 (58.01)	614 (60.31)	1.000 (reference)		
A-C	217 (28.04)	285 (28.00)	0.960 (0.775 - 1.190)	0.712	0.712
G-G	3 (0.39)	26 (2.55)	6.338 (1.906 - 21.070)	**0.0004**	**0.001**
G-C	105 (13.57)	93 (9.14)	0.648 (0.478 - 0.878)	**0.005**	**0.007**
SPARCL1 rs7695558 A>G/rs1130643 T>C					
A-T	657 (84.88)	875 (85.95)	1.000 (reference)		
A-C	9 (1.16)	24 (2.36)	2.002 (0.924 - 4.337)	0.073	0.090
G-T	18 (2.33)	39 (3.83)	1.627 (0.922 - 2.870)	0.090	0.090
G-C	90 (11.63)	80 (7.86)	0.667 (0.486 - 0.917)	0.012	**0.036**
SPARCL1 rs1049544 G>C/rs1130643 T>C					
G-T	448 (57.88)	634 (62.28)	1.000 (reference)		
G-C	4 (0.52)	6 (0.59)	1.060 (0.297 - 3.779)	0.929	0.929
C-T	227 (29.33)	280 (27.50)	0.872 (0.705 - 1.078)	0.206	0.309
C-C	95 (12.27)	98 (9.63)	0.729 (0.536 - 0.991)	**0.043**	0.129

OR, odds ratio; 95% CI, 95% confidence interval; *SPARCL1*, secreted protein acidic and rich in cysteine-like 1. Bold values indicate statistical significance ($p < 0.05$ or FDR-$p < 0.05$).

Table S3. Combined genotype analysis for *SPARCL1* polymorphisms in ischemic stroke patients and controls (Set 1)

Genotype	Controls (n = 387)	Patients (n = 509)	AOR (95% CI)[a]	*P*	*FDR-P*
SPARCL1 rs1049539 T>C/rs7695558 A>G/rs1049544 G>C					
TT/AA/GG	106 (27.39)	143 (28.09)	1.000 (reference)		
TT/AA/GC	28 (7.24)	31 (6.09)	0.760 (0.419 - 1.381)	0.368	0.841
TT/AA/CC	2 (0.52)	8 (1.57)	2.924 (0.585 - 14.616)	0.191	0.611
TT/AG/GG	1 (0.26)	9 (1.77)	5.801 (0.691 - 48.661)	0.105	0.420
TT/AG/GC	40 (10.34)	47 (9.23)	0.989 (0.590 - 1.657)	0.965	0.965
TT/AG/CC	8 (2.07)	2 (0.39)	0.117 (0.021 - 0.634)	**0.013**	0.208
TT/GG/GG	0 (0.0)	0 (0.0)	N/A	N/A	N/A
TT/GG/GC	0 (0.0)	3 (0.59)	N/A	N/A	N/A
TT/GG/CC	9 (2.33)	4 (0.79)	0.276 (0.077 - 0.993)	**0.049**	0.261
TC/AA/GG	26 (6.72)	33 (6.48)	0.877 (0.477 - 1.615)	0.674	0.942
TC/AA/GC	90 (23.26)	139 (27.31)	1.075 (0.736 - 1.571)	0.707	0.942
TC/AA/CC	10 (2.58)	14 (2.75)	1.130 (0.461 - 2.770)	0.790	0.965
TC/AG/GG	0 (0.0)	4 (0.79)	N/A	N/A	N/A
TC/AG/GC	16 (4.13)	21 (4.13)	1.074 (0.516 - 2.234)	0.849	0.965
TC/AG/CC	19 (4.91)	12 (2.36)	0.425 (0.187 - 0.968)	**0.042**	0.261
TC/GG/GG	0 (0.0)	1 (0.2)	N/A	N/A	N/A
TC/GG/GC	1 (0.26)	0 (0.0)	N/A	N/A	N/A
TC/GG/CC	0 (0.0)	3 (0.59)	N/A	N/A	N/A
CC/AA/GG	1 (0.26)	3 (0.59)	2.036 (0.168 - 24.600)	0.576	0.926
CC/AA/GC	6 (1.55)	12 (2.36)	1.353 (0.466 - 3.926)	0.579	0.926
CC/AA/CC	20 (5.17)	18 (3.54)	0.755 (0.367 - 1.556)	0.447	0.894
CC/AG/GG	0 (0.0)	0 (0.0)	N/A	N/A	N/A
CC/AG/GC	3 (0.78)	1 (0.2)	0.327 (0.032 - 3.392)	0.349	0.841
CC/AG/CC	1 (0.26)	1 (0.2)	1.117 (0.067 - 18.743)	0.939	0.965
CC/GG/GG	0 (0.0)	0 (0.0)	N/A	N/A	N/A
CC/GG/GC	0 (0.0)	0 (0.0)	N/A	N/A	N/A
CC/GG/CC	0 (0.0)	0 (0.0)	N/A	N/A	N/A
SPARCL1 rs1049539 T>C/rs7695558 A>G/rs1130643 T>C					
TT/AA/TT	133 (34.37)	168 (33.01)	1.000 (reference)		
TT/AA/TC	3 (0.78)	12 (2.36)	3.518 (0.945 - 13.091)	0.061	0.366
TT/AA/CC	0 (0.0)	2 (0.39)	N/A	N/A	N/A
TT/AG/TT	2 (0.52)	11 (2.16)	4.836 (1.009 - 23.173)	**0.049**	0.366
TT/AG/TC	47 (12.14)	47 (9.23)	0.817 (0.504 - 1.325)	0.413	0.756
TT/AG/CC	0 (0.0)	0 (0.0)	N/A	N/A	N/A
TT/GG/TT	0 (0.0)	0 (0.0)	N/A	N/A	N/A
TT/GG/TC	2 (0.52)	3 (0.59)	1.577 (0.255 - 9.757)	0.624	0.867
TT/GG/CC	7 (1.81)	4 (0.79)	0.401 (0.106 - 1.514)	0.178	0.570
TC/AA/TT	122 (31.52)	179 (35.17)	1.142 (0.815 - 1.600)	0.441	0.756
TC/AA/TC	4 (1.03)	7 (1.38)	1.149 (0.311 - 4.243)	0.835	0.867
TC/AA/CC	0 (0.0)	0 (0.0)	N/A	N/A	N/A
TC/AG/TT	11 (2.84)	19 (3.73)	1.478 (0.663 - 3.296)	0.339	0.756
TC/AG/TC	24 (6.20)	18 (3.54)	0.629 (0.314 - 1.259)	0.190	0.570
TC/AG/CC	0 (0.0)	0 (0.0)	N/A	N/A	N/A
TC/GG/TT	0 (0.0)	1 (0.2)	N/A	N/A	N/A
TC/GG/TC	1 (0.26)	1 (0.2)	0.662 (0.038 - 11.460)	0.777	0.867
TC/GG/CC	0 (0.0)	2 (0.39)	N/A	N/A	N/A
CC/AA/TT	26 (6.72)	33 (6.48)	1.052 (0.582 - 1.902)	0.867	0.867
CC/AA/TC	0 (0.0)	0 (0.0)	N/A	N/A	N/A
CC/AA/CC	1 (0.26)	0 (0.0)	N/A	N/A	N/A
CC/AG/TT	2 (0.52)	2 (0.39)	1.253 (0.170 - 9.260)	0.825	0.867
CC/AG/TC	2 (0.52)	0 (0.0)	N/A	N/A	N/A
CC/AG/CC	0 (0.0)	0 (0.0)	N/A	N/A	N/A
CC/GG/TT	0 (0.0)	0 (0.0)	N/A	N/A	N/A
CC/GG/TC	0 (0.0)	0 (0.0)	N/A	N/A	N/A
CC/GG/CC	0 (0.0)	0 (0.0)	N/A	N/A	N/A
SPARCL1 rs1049539 T>C/rs1049544 G>C/rs1130643 T>C					
TT/GG/TT	105 (27.13)	150 (29.47)	1.000 (reference)		
TT/GG/TC	2 (0.52)	1 (0.2)	0.280 (0.019 - 4.149)	0.355	0.761
TT/GG/CC	0 (0.0)	1 (0.2)	N/A	N/A	N/A
TT/GC/TT	28 (7.24)	23 (4.52)	0.531 (0.281 - 1.005)	0.052	0.390
TT/GC/TC	40 (10.34)	58 (11.39)	1.134 (0.690 - 1.864)	0.619	0.785
TT/GC/CC	0 (0.0)	0 (0.0)	N/A	N/A	N/A
TT/CC/TT	2 (0.52)	6 (1.18)	1.983 (0.377 - 10.435)	0.419	0.785
TT/CC/TC	10 (2.58)	3 (0.59)	0.149 (0.036 - 0.610)	**0.008**	0.120
TT/CC/CC	7 (1.81)	5 (0.98)	0.469 (0.135 - 1.630)	0.234	0.761
TC/GG/TT	25 (6.46)	37 (7.27)	1.037 (0.570 - 1.886)	0.906	0.906
TC/GG/TC	1 (0.26)	1 (0.2)	0.496 (0.029 - 8.475)	0.628	0.785
TC/GG/CC	0 (0.0)	0 (0.0)	N/A	N/A	N/A
TC/GC/TT	96 (24.81)	151 (29.67)	1.047 (0.723 - 1.517)	0.808	0.865
TC/GC/TC	11 (2.84)	9 (1.77)	0.559 (0.213 - 1.463)	0.236	0.761
TC/GC/CC	0 (0.0)	0 (0.0)	N/A	N/A	N/A
TC/CC/TT	12 (3.10)	11 (2.16)	0.640 (0.260 - 1.575)	0.331	**0.020**
TC/CC/TC	17 (4.39)	16 (3.14)	0.665 (0.307 - 1.439)	0.300	0.761
TC/CC/CC	0 (0.0)	2 (0.39)	N/A	N/A	N/A
CC/GG/TT	1 (0.26)	3 (0.59)	1.948 (0.164 - 23.204)	0.598	0.785
CC/GG/TC	0 (0.0)	0 (0.0)	N/A	N/A	N/A
CC/GG/CC	0 (0.0)	0 (0.0)	N/A	N/A	N/A
CC/GC/TT	8 (2.07)	13 (2.55)	1.147 (0.436 - 3.020)	0.781	**0.043**
CC/GC/TC	1 (0.26)	0 (0.0)	N/A	N/A	N/A
CC/GC/CC	0 (0.0)	0 (0.0)	N/A	N/A	N/A
CC/CC/TT	19 (4.91)	19 (3.73)	0.798 (0.389 - 1.637)	0.539	0.785
CC/CC/TC	1 (0.26)	0 (0.0)	N/A	N/A	N/A
CC/CC/CC	1 (0.26)	0 (0.0)	N/A	N/A	N/A

AOR, adjusted odds ratio; 95% CI, 95% confidence interval; *SPARCL1*, secreted protein acidic and rich in cysteine-like 1. [a] Adjusted for age, sex, hypertension, diabetes mellitus, hyperlipidemia, and smoking. Bold values indicate statistical significance ($p < 0.05$ or FDR-$p < 0.05$).

Table S4. Combined genotype analysis for *SPARCL1* polymorphisms in ischemic stroke patients and controls (Set 2)

Genotype	Controls (*n* = 387)	Patients (*n* = 509)	AOR (95% CI)[a]	*P*	*FDR-P*
SPARCL1 rs7695558 A>G/rs1049544 G>C/rs1130643 T>C					
AA/GG/TT	131 (33.85)	176 (34.58)	1.000 (reference)		
AA/GG/TC	2 (0.52)	2 (0.39)	0.874 (0.109 - 7.031)	0.899	0.965
AA/GG/CC	0 (0.0)	1 (0.2)	N/A	N/A	N/A
AA/GC/TT	120 (31.01)	169 (33.2)	0.970 (0.691 - 1.363)	0.861	0.965
AA/GC/TC	4 (1.03)	13 (2.55)	2.257 (0.686 - 7.430)	0.181	0.750
AA/GC/CC	0 (0.0)	0 (0.0)	N/A	N/A	N/A
AA/CC/TT	30 (7.75)	35 (6.88)	0.987 (0.558 - 1.748)	0.965	0.965
AA/CC/TC	1 (0.26)	4 (0.79)	3.833 (0.401 - 36.607)	0.243	0.750
AA/CC/CC	1 (0.26)	1 (0.2)	1.420 (0.085 - 23.853)	0.808	0.965
AG/GG/TT	0 (0.0)	13 (2.55)	N/A	N/A	N/A
AG/GG/TC	1 (0.26)	0 (0.0)	N/A	N/A	N/A
AG/GG/CC	0 (0.0)	0 (0.0)	N/A	N/A	N/A
AG/GC/TT	12 (3.10)	18 (3.54)	1.328 (0.591 - 2.984)	0.493	0.801
AG/GC/TC	47 (12.14)	51 (10.02)	0.948 (0.583 - 1.542)	0.830	0.965
AG/GC/CC	0 (0.0)	0 (0.0)	N/A	N/A	N/A
AG/CC/TT	3 (0.78)	1 (0.2)	0.203 (0.020 - 2.070)	0.178	0.750
AG/CC/TC	25 (6.46)	14 (2.75)	0.348 (0.161 - 0.749)	**0.007**	0.091
AG/CC/CC	0 (0.0)	0 (0.0)	N/A	N/A	N/A
GG/GG/TT	0 (0.0)	1 (0.2)	N/A	N/A	N/A
GG/GG/TC	0 (0.0)	0 (0.0)	N/A	N/A	N/A
GG/GG/CC	0 (0.0)	0 (0.0)	N/A	N/A	N/A
GG/GC/TT	0 (0.0)	0 (0.0)	N/A	N/A	N/A
GG/GC/TC	1 (0.26)	3 (0.59)	3.017 (0.303 - 30.023)	0.346	0.750
GG/GC/CC	0 (0.0)	0 (0.0)	N/A	N/A	N/A
GG/CC/TT	0 (0.0)	0 (0.0)	N/A	N/A	N/A
GG/CC/TC	2 (0.52)	1 (0.2)	0.275 (0.023 - 3.297)	0.308	0.750
GG/CC/CC	7 (1.81)	6 (1.18)	0.646 (0.197 - 2.122)	0.471	0.801
SPARCL1 rs1049539 T>C/rs7695558 A>G					
TT/AA	136 (35.14)	182 (35.76)	1.000 (reference)		
TT/AG	49 (12.66)	58 (11.39)	0.910 (0.574 - 1.442)	0.689	0.804
TT/GG	9 (2.33)	7 (1.38)	0.601 (0.209 - 1.726)	0.344	0.804
TC/AA	126 (32.56)	186 (36.54)	1.081 (0.777 - 1.503)	0.645	0.804
TC/AG	35 (9.04)	37 (7.27)	0.840 (0.490 - 1.440)	0.526	0.804
TC/GG	1 (0.26)	4 (0.79)	2.815 (0.301 - 26.315)	0.364	0.804
CC/AA	27 (6.98)	33 (6.48)	0.956 (0.533 - 1.717)	0.881	0.804
CC/AG	4 (1.03)	2 (0.39)	0.484 (0.084 - 2.780)	0.416	0.881
CC/GG	0 (0.0)	0 (0.0)	N/A	N/A	N/A
SPARCL1 rs1049539 T>C/rs1049544 G>C					
TT/GG	107 (27.65)	152 (29.86)	1.000 (reference)		
TT/GC	68 (17.57)	81 (15.91)	0.872 (0.571 - 1.334)	0.528	0.949
TT/CC	19 (4.91)	14 (2.75)	0.455 (0.211 - 0.980)	**0.044**	0.352
TC/GG	26 (6.72)	38 (7.47)	1.023 (0.569 - 1.838)	0.940	0.997
TC/GC	107 (27.65)	160 (31.43)	0.997 (0.694 - 1.431)	0.986	0.997
TC/CC	29 (7.49)	29 (5.7)	0.724 (0.397 - 1.319)	0.291	0.949
CC/GG	1 (0.26)	3 (0.59)	1.956 (0.168 - 22.824)	0.593	0.949
CC/GC	9 (2.33)	13 (2.55)	1.002 (0.395 - 2.538)	0.997	0.997
CC/CC	21 (5.43)	19 (3.73)	0.734 (0.364 - 1.479)	0.387	0.949
SPARCL1 rs1049539 T>C/rs1130643 T>C					
TT/TT	135 (34.88)	179 (35.17)	1.000 (reference)		
TT/TC	52 (13.44)	62 (12.18)	0.939 (0.599 - 1.471)	0.782	0.954
TT/CC	7 (1.81)	6 (1.18)	0.609 (0.188 - 1.975)	0.409	0.890
TC/TT	133 (34.37)	199 (39.1)	1.109 (0.800 - 1.537)	0.534	0.890
TC/TC	29 (7.49)	26 (5.11)	0.676 (0.368 - 1.243)	0.207	0.890
TC/CC	0 (0.0)	2 (0.39)	N/A	N/A	N/A
CC/TT	28 (7.24)	35 (6.88)	1.017 (0.572 - 1.808)	0.954	0.954
CC/TC	2 (0.52)	0 (0.0)	N/A	N/A	N/A
CC/CC	1 (0.26)	0 (0.0)	N/A	N/A	N/A
SPARCL1 rs7695558 A>G/rs1049544 G>C					
AA/GG	133 (34.37)	179 (35.17)	1.000 (reference)		
AA/GC	124 (32.04)	182 (35.76)	0.999 (0.715 - 1.397)	0.996	0.996
AA/CC	32 (8.27)	40 (7.86)	1.085 (0.627 - 1.877)	0.771	0.996
AG/GG	1 (0.26)	13 (2.55)	10.995(1.370 - 88.261)	**0.024**	0.084
AG/GC	59 (15.25)	69 (13.56)	1.018 (0.653 - 1.586)	0.937	0.996
AG/CC	28 (7.24)	15 (2.95)	0.328 (0.158 - 0.683)	**0.003**	**0.021**
GG/GG	0 (0.0)	1 (0.2)	N/A	N/A	
GG/GC	1 (0.26)	3 (0.59)	3.087 (0.311 - 30.638)	0.336	0.588
GG/CC	9 (2.33)	7 (1.38)	0.544 (0.185 - 1.598)	0.268	0.588
SPARCL1 rs7695558 A>G/rs1130643 T>C					
AA/TT	281 (72.61)	380 (74.66)	1.000 (reference)		
AA/TC	7 (1.81)	19 (3.73)	2.087 (0.835 - 5.215)	0.116	0.292
AA/CC	1 (0.26)	2 (0.39)	2.028 (0.169 - 24.313)	0.577	0.692
AG/TT	15 (3.88)	32 (6.29)	1.633 (0.843 - 3.164)	0.146	0.292
AG/TC	73 (18.86)	65 (12.77)	0.679 (0.461 - 1.001)	0.050	0.292
AG/CC	0 (0.0)	0 (0.0)	N/A	N/A	N/A
GG/TT	0 (0.0)	1 (0.2)	N/A	N/A	
GG/TC	3 (0.78)	4 (0.79)	1.216 (0.258 - 5.741)	0.805	0.805
GG/CC	7 (1.81)	6 (1.18)	0.679 (0.216 - 2.131)	0.507	0.692
SPARCL1 rs1049544 G>C/rs1130643 T>C					
GG/TT	131 (33.85)	190 (37.33)	1.000 (reference)		
GG/TC	3 (0.78)	2 (0.39)	0.356 (0.049 - 2.612)	0.310	0.696
GG/CC	0 (0.0)	1 (0.2)	N/A	N/A	N/A
GC/TT	132 (34.11)	187 (36.74)	0.911 (0.656 - 1.266)	0.580	0.696
GC/TC	52 (13.44)	67 (13.16)	0.986 (0.629 - 1.545)	0.951	0.951
GC/CC	0 (0.0)	0 (0.0)	N/A	N/A	N/A

CC/TT	33 (8.53)	36 (7.07)	0.823 (0.475 - 1.427)	0.488	0.696
CC/TC	28 (7.24)	19 (3.73)	0.421 (0.214 - 0.826)	**0.012**	0.072
CC/CC	8 (2.07)	7 (1.38)	0.678 (0.226 - 2.032)	0.488	0.696

AOR, adjusted odds ratio; 95% CI, 95% confidence interval; *SPARCL1*, secreted protein acidic and rich in cysteine-like 1. [a] Adjusted for age, sex, hypertension, diabetes mellitus, hyperlipidemia, and smoking. Bold values indicate statistical significance ($p < 0.05$ or FDR-$p < 0.05$).

Table S5. Differences of clinical variables in ischemic stroke patients and controls stratified by *SPARCL1* polymorphism status by ANOVA

Genotypes	Age (years)	BMI (kg/m2)	HDL-C (mg/dl)	Homocysteine (μmol/L)	Folate (nmol/L)	Vitamin B12 (pg/mL)	Total cholesterol (mg/dL)	Triglyceride (mg/dL)	PLT (103/μL)	PT (s)	aPTT (s)	Fibrinogen (mg/dL)	Antithrombin (%)	BUN (mg/dL)	Uric acid (mg/dL)
	**Mean ± SD**	**Mean ± SD**	**Mean ± SD**	**Mean ± SD**	**Mean ± SD**	**Mean ± SD**	**Mean ± SD**	**Mean ± SD**	**Mean ± SD**	**Mean ± SD**	**Mean ± SD**	**Mean ± SD**	**Mean ± SD**	**Mean ± SD**	**Mean ± SD**
***SPARCL1* rs1049539 T>C**															
TT	63.333 ± 11.456	24.461 ± 4.062	46.053 ± 17.107	10.493 ± 4.744	7.630 ± 6.965	693.391 ± 311.729	192.575 ± 38.920	142.097 ± 78.585	243.904 ± 68.339	11.702 ± 0.736	31.062 ± 7.184	426.693 ± 133.009	93.394 ± 17.080	16.149 ± 6.161	4.675 ± 1.528
TC	63.733 ± 10.257	23.862 ± 3.150	44.323 ± 13.316	11.072 ± 6.720	7.810 ± 6.378	682.807 ± 298.081	190.695 ± 41.182	158.743 ± 125.198	246.112 ± 91.238	12.104 ± 3.994	31.355 ± 5.892	417.683 ± 124.847	92.893 ± 33.591	16.198 ± 5.280	4.683 ± 1.504
CC	63.045 ± 11.584	24.697 ± 2.776	44.918 ± 11.565	10.005 ± 4.676	8.035 ± 4.934	697.631 ± 286.674	185.600 ± 34.245	134.954 ± 77.271	238.833 ± 66.041	11.706 ± 0.596	31.010 ± 5.656	399.278 ± 127.709	90.746 ± 21.527	15.423 ± 5.610	4.642 ± 1.290
P^a	0.759	**0.048**	0.625	0.208	0.776	0.929	0.489	0.186	0.788	0.164	0.870	0.238	0.851	0.725	0.963
***SPARCL1* rs7695558 A>G**															
AA	63.658 ± 10.748	24.060 ± 3.179	45.280 ± 16.119	10.626 ± 5.429	7.608 ± 5.613	691.308 ± 302.550	191.686 ± 40.344	150.230 ± 106.150	245.196 ± 80.700	11.901 ± 3.053	30.963 ± 5.912	422.634 ± 125.822	92.271 ± 27.948	16.055 ± 5.370	4.647 ± 1.509
AG	62.541 ± 11.712	24.945 ± 4.944	44.515 ± 11.680	11.001 ± 6.754	8.281 ± 9.497	688.736 ± 313.868	187.219 ± 35.487	140.596 ± 82.608	241.208 ± 72.699	11.794 ± 0.772	31.869 ± 7.358	412.039 ± 140.405	95.528 ± 16.368	16.321 ± 7.035	4.776 ± 1.447
GG	66.143 ± 10.399	23.056 ± 2.574	49.356 ± 12.545	10.809 ± 3.275	7.164 ± 3.698	624.619 ± 258.363	211.905 ± 44.133	174.857 ± 106.332	249.905 ± 73.674	11.847 ± 1.002	32.281 ± 14.085	446.746 ± 136.769	94.100 ± 17.160	16.362 ± 5.618	4.750 ± 1.680
P^a	0.202	**0.024**	0.479	0.698	0.454	0.611	**0.016**	0.212	0.760	0.886	0.226	0.549	0.444	0.851	0.475
***SPARCL1* rs1049544 G>C**															
GG	63.520 ± 10.571	24.079 ± 3.095	45.981 ± 18.648	10.588 ± 4.216	7.340 ± 4.689	665.538 ± 280.130	192.166 ± 37.859	145.669 ± 86.877	242.804 ± 69.272	11.801 ± 1.117	31.133 ± 6.857	425.748 ± 126.803	91.912 ± 16.902	16.078 ± 5.352	4.676 ± 1.495
GC	63.395 ± 11.366	24.346 ± 4.167	44.410 ± 13.003	10.936 ± 6.764	8.048 ± 8.016	698.178 ± 310.975	189.722 ± 41.139	151.536 ± 114.067	244.553 ± 89.901	11.947 ± 3.683	31.052 ± 6.047	419.898 ± 133.418	93.860 ± 31.929	16.202 ± 6.039	4.656 ± 1.571
CC	63.702 ± 10.540	24.180 ± 2.764	46.265 ± 11.831	10.241 ± 4.865	7.684 ± 5.052	717.264 ± 332.835	194.016 ± 38.696	147.543 ± 92.541	248.389 ± 60.258	11.823 ± 0.778	31.786 ± 7.312	410.298 ± 117.707	92.615 ± 17.032	15.934 ± 5.763	4.744 ± 1.260
P^a	0.920	0.645	0.390	0.453	0.372	0.169	0.560	0.703	0.788	0.734	0.586	0.710	0.647	0.871	0.713
***SPARCL1* rs1130643 T>C**															
TT	63.481 ± 10.789	24.102 ± 3.189	45.133 ± 15.862	10.698 ± 5.609	7.613 ± 5.608	693.126 ± 300.330	191.067 ± 40.044	148.630 ± 105.376	243.922 ± 79.351	11.915 ± 3.009	31.020 ± 6.059	422.016 ± 125.977	92.617 ± 27.648	16.013 ± 5.344	4.649 ± 1.487
TC	63.345 ± 11.658	24.825 ± 5.048	45.197 ± 12.282	10.776 ± 6.166	8.347 ± 9.710	674.857 ± 322.472	190.583 ± 36.629	148.375 ± 86.807	246.172 ± 77.775	11.731 ± 0.781	31.600 ± 6.993	415.458 ± 141.252	94.850 ± 15.858	16.488 ± 7.174	4.815 ± 1.564
CC	65.188 ± 10.864	22.830 ± 2.246	50.130 ± 12.860	10.396 ± 3.378	6.683 ± 4.150	667.600 ± 257.315	206.600 ± 50.093	162.067 ± 89.407	251.375 ± 74.529	11.800 ± 1.052	33.893 ± 15.597	428.837 ± 157.296	90.079 ± 18.237	16.788 ± 6.378	4.400 ± 1.368
P^a	0.972	**0.014**	0.618	0.980	0.296	0.684	0.288	0.838	0.858	0.751	0.320	0.875	0.745	0.589	0.503

ANOVA, analysis of variance; SD, standard deviation; BMI, body mass index; HDL-C, high-density lipoprotein cholesterol; PLT, platelet; PT, prothrombin time; aPTT, activated partial thromboplastin time; BUN, blood urea nitrogen; *SPARCL1*, secreted protein acidic and rich in cysteine-like 1. [a] *p*-value was calculated using ANOVA. Bold values indicate statistical significance ($p < 0.05$).

Table S6. Differences of clinical variables in controls stratified by *SPARCL1* polymorphism status by ANOVA

**Genotypes**	**Age (years)**	**BMI (kg/m2)**	**HDL-C (mg/dl)**	**Homocysteine (μmol/L)**	**Folate (nmol/L)**	**Vitamin B12 (pg/mL)**	**Total cholesterol (mg/dL)**	**Triglyceride (mg/dL)**	**PLT (103/μL)**	**PT (s)**	**aPTT (s)**	**Fibrinogen (mg/dL)**	**Antithrombin (%)**	**BUN (mg/dL)**	**Uric acid (mg/dL)**
	**Mean ± SD**	**Mean ± SD**	**Mean ± SD**	**Mean ± SD**	**Mean ± SD**	**Mean ± SD**	**Mean ± SD**	**Mean ± SD**	**Mean ± SD**	**Mean ± SD**	**Mean ± SD**	**Mean ± SD**	**Mean ± SD**	**Mean ± SD**	**Mean ± SD**
***SPARCL1* rs1049539 T>C**															
TT	62.716 ± 11.170	24.451 ± 3.201	46.461 ± 15.372	10.273 ± 4.397	8.566 ± 8.842	703.995 ± 286.788	195.197 ± 38.650	142.617 ± 74.943	244.582 ± 64.850	11.687 ± 0.721	32.023 ± 10.399	385.299 ± 112.338	92.171 ± 17.553	15.943 ± 4.796	4.689 ± 1.419
TC	63.364 ± 9.855	23.835 ± 3.221	46.129 ± 12.016	9.851 ± 3.860	9.410 ± 7.602	679.723 ± 278.298	191.440 ± 37.753	153.855 ± 104.036	245.698 ± 68.480	11.919 ± 0.862	32.602 ± 7.617	420.486 ± 138.517	98.803 ± 68.608	16.046 ± 4.960	4.772 ± 1.563
CC	60.548 ± 11.809	24.967 ± 3.130	48.950 ± 11.709	9.653 ± 3.251	8.726 ± 5.549	622.000 ± 199.711	187.967 ± 32.149	122.833 ± 57.003	233.194 ± 55.481	11.672 ± 0.591	32.698 ± 7.877	360.420 ± 79.150	89.373 ± 20.981	16.377 ± 6.541	4.633 ± 1.162
P^a	0.231	0.129	0.647	0.385	0.795	0.531	0.647	0.877	0.651	0.278	0.902	0.493	0.680	0.734	0.820
***SPARCL1* rs7695558 A>G**															
AA	63.104 ± 10.046	24.152 ± 3.130	45.841 ± 14.520	10.142 ± 4.196	8.659 ± 6.225	686.196 ± 274.047	194.089 ± 38.202	147.149 ± 88.730	244.124 ± 59.950	11.775 ± 0.764	31.978 ± 8.213	410.327 ± 125.328	94.056 ± 52.074	16.037 ± 5.096	4.743 ± 1.478
AG	61.898 ± 12.822	24.640 ± 3.433	49.078 ± 12.063	9.700 ± 3.884	9.912 ± 12.655	705.941 ± 295.857	188.640 ± 33.797	138.686 ± 83.686	242.314 ± 82.319	11.822 ± 0.852	32.607 ± 9.638	366.724 ± 112.734	95.296 ± 14.777	16.091 ± 4.914	4.706 ± 1.358
GG	62.500 ± 8.462	22.725 ± 3.211	40.840 ± 8.415	10.359 ± 2.850	8.137 ± 4.247	555.400 ± 173.242	201.300 ± 55.968	168.400 ± 90.037	259.500 ± 63.172	11.607 ± 0.469	39.450 ± 20.904	355.000 ± 48.218	100.000 ± 2.828	14.980 ± 3.446	4.133 ± 1.888
P^a	0.506	0.495	0.290	0.719	0.458	0.274	0.286	0.401	0.714	0.815	0.097	0.147	0.975	0.801	0.476
***SPARCL1* rs1049544 G>C**															
GG	63.216 ± 9.615	24.069 ± 2.990	46.075 ± 17.324	10.254 ± 3.967	8.200 ± 4.662	690.395 ± 302.105	194.277 ± 36.088	139.692 ± 77.027	248.446 ± 62.370	11.728 ± 0.702	32.490 ± 10.079	406.952 ± 123.313	87.708 ± 16.830	15.871 ± 4.998	4.682 ± 1.352
GC	62.179 ± 11.955	24.351 ± 3.440	46.607 ± 11.531	10.049 ± 4.565	9.536 ± 10.447	687.624 ± 259.043	192.732 ± 39.004	153.575 ± 94.728	239.778 ± 72.408	11.804 ± 0.831	32.201 ± 8.393	401.154 ± 127.956	100.234 ± 58.194	16.110 ± 4.730	4.742 ± 1.590
CC	63.725 ± 9.000	24.377 ± 3.035	47.105 ± 11.763	9.639 ± 2.827	8.708 ± 5.763	679.941 ± 279.010	191.471 ± 38.125	136.912 ± 86.300	247.290 ± 51.626	11.830 ± 0.792	32.334 ± 9.089	349.250 ± 75.004	88.125 ± 20.360	16.077 ± 5.775	4.731 ± 1.319
P^a	0.784	0.833	0.976	0.669	0.382	0.984	0.762	0.233	0.486	0.592	0.968	0.363	0.232	0.983	0.863
***SPARCL1* rs1130643 T>C**															
TT	62.764 ± 10.155	24.202 ± 3.204	46.231 ± 14.417	10.199 ± 4.338	8.642 ± 6.226	686.254 ± 274.110	193.198 ± 38.054	145.146 ± 87.281	243.083 ± 58.286	11.801 ± 0.779	32.132 ± 8.514	409.230 ± 122.313	94.907 ± 50.617	16.059 ± 5.107	4.736 ± 1.455
TC	62.988 ± 12.645	24.391 ± 3.293	47.639 ± 12.289	9.551 ± 3.192	10.021 ± 12.905	704.150 ± 298.534	191.432 ± 34.000	147.469 ± 89.664	245.222 ± 87.388	11.756 ± 0.807	32.191 ± 8.808	360.737 ± 118.559	92.998 ± 14.881	15.894 ± 4.786	4.766 ± 1.500
CC	62.875 ± 9.047	25.150 ± 2.475	42.967 ± 8.893	9.633 ± 2.913	8.288 ± 4.786	550.875 ± 105.863	203.500 ± 62.548	151.625 ± 86.619	270.500 ± 64.737	11.433 ± 0.489	40.258 ± 22.730	372.500 ± 53.033	98.000 ± 0.000	15.950 ± 4.166	3.675 ± 0.950
P^a	0.518	0.384	0.708	0.390	0.328	0.136	0.649	0.939	0.442	0.526	0.869	0.085	0.934	0.840	0.686

ANOVA, analysis of variance; SD, standard deviation; BMI, body mass index; HDL-C, high-density lipoprotein cholesterol; PLT, platelet; PT, prothrombin time; aPTT, activated partial thromboplastin time; BUN, blood urea nitrogen; *SPARCL1*, secreted protein acidic and rich in cysteine-like 1. [a] *p*-value was calculated using ANOVA. Bold values indicate statistical significance ($p < 0.05$).

Table S7. Differences of clinical variables in ischemic stroke patients stratified by *SPARCL1* polymorphism status by ANOVA

Genotypes	Age (years)	BMI (kg/m2)	HDL-C (mg/dl)	Homocysteine (μmol/L)	Folate (nmol/L)	Vitamin B12 (pg/mL)	Total cholesterol (mg/dL)	Triglyceride (mg/dL)	PLT (10^3/μL)	PT (s)	aPTT (s)	Fibrinogen (mg/dL)	Antithrombin (%)	BUN (mg/dL)	Uric acid (mg/dL)
	**Mean ± SD**	**Mean ± SD**	**Mean ± SD**	**Mean ± SD**	**Mean ± SD**	**Mean ± SD**	**Mean ± SD**	**Mean ± SD**	**Mean ± SD**	**Mean ± SD**	**Mean ± SD**	**Mean ± SD**	**Mean ± SD**	**Mean ± SD**	**Mean ± SD**
***SPARCL1* rs1049539 T>C**															
TT	63.818 ± 11.676	24.467 ± 4.472	45.898 ± 17.749	10.666 ± 5.003	6.896 ± 4.925	685.144 ± 330.177	190.547 ± 39.085	141.695 ± 81.442	243.385 ± 71.019	11.710 ± 0.746	30.523 ± 4.410	440.247 ± 136.628	93.784 ± 16.947	16.308 ± 7.043	4.665 ± 1.607
TC	63.996 ± 10.548	23.875 ± 3.125	43.780 ± 13.663	11.932 ± 8.052	6.669 ± 5.054	684.991 ± 311.934	190.169 ± 43.517	162.229 ± 138.450	246.403 ± 104.469	12.200 ± 4.886	30.706 ± 4.643	416.986 ± 121.559	91.422 ± 15.665	16.306 ± 5.504	4.621 ± 1.463
CC	65.257 ± 11.081	24.515 ± 2.546	43.732 ± 11.425	10.317 ± 5.679	7.424 ± 4.306	766.588 ± 335.934	183.571 ± 36.288	145.343 ± 90.702	243.829 ± 74.615	11.729 ± 0.608	29.901 ± 3.207	411.813 ± 138.567	91.249 ± 22.055	14.553 ± 4.528	4.650 ± 1.410
P^a	0.775	0.246	0.330	0.081	0.675	0.365	0.636	0.360	0.931	0.263	0.600	0.138	0.307	0.288	0.954
***SPARCL1* rs7695558 A>G**															
AA	64.057 ± 11.223	24.016 ± 3.205	45.104 ± 16.603	10.975 ± 6.148	6.851 ± 4.999	694.997 ± 321.852	189.977 ± 41.765	152.433 ± 117.088	245.955 ± 92.683	11.966 ± 3.712	30.446 ± 4.207	425.820 ± 125.923	91.800 ± 16.606	16.068 ± 5.564	4.580 ± 1.529
AG	63.124 ± 10.639	25.168 ± 5.815	42.573 ± 11.014	12.168 ± 8.399	6.819 ± 4.889	673.660 ± 329.631	185.959 ± 37.051	142.289 ± 82.037	240.227 ± 63.387	11.773 ± 0.712	31.353 ± 5.210	431.387 ± 147.028	95.622 ± 17.050	16.526 ± 8.505	4.837 ± 1.526
GG	69.455 ± 11.246	23.188 ± 2.463	53.227 ± 12.450	11.218 ± 3.709	6.279 ± 3.052	687.545 ± 312.000	221.545 ± 29.351	180.727 ± 123.439	241.182 ± 84.192	12.000 ± 1.228	27.718 ± 4.022	474.270 ± 144.142	92.920 ± 18.701	17.618 ± 6.985	5.255 ± 1.374
P^a	0.197	**0.030**	0.073	0.280	0.931	0.844	**0.023**	0.487	0.839	0.876	**0.020**	0.493	0.150	0.606	0.136
***SPARCL1* rs1049544 G>C**															
GG	63.731 ± 11.206	24.084 ± 3.154	45.950 ± 19.108	10.821 ± 4.377	6.747 ± 4.628	648.390 ± 263.374	190.721 ± 39.053	149.758 ± 92.995	238.984 ± 73.493	11.839 ± 1.280	30.436 ± 4.238	430.447 ± 127.571	93.041 ± 16.789	16.220 ± 5.591	4.673 ± 1.587
GC	64.276 ± 10.859	24.344 ± 4.494	43.671 ± 13.402	11.576 ± 7.929	6.964 ± 5.390	705.754 ± 343.762	187.583 ± 42.537	150.076 ± 126.273	247.937 ± 100.474	12.022 ± 4.502	30.454 ± 4.256	425.934 ± 134.839	91.905 ± 16.981	16.267 ± 6.832	4.595 ± 1.558
CC	63.677 ± 12.102	24.046 ± 2.581	45.947 ± 11.944	10.901 ± 6.358	6.561 ± 3.878	758.869 ± 382.180	196.852 ± 39.443	159.393 ± 98.398	249.613 ± 69.025	11.819 ± 0.772	31.361 ± 5.608	428.387 ± 122.421	93.921 ± 15.912	15.776 ± 5.793	4.758 ± 1.203
P^a	0.852	0.749	0.279	0.454	0.810	**0.039**	0.267	0.824	0.507	0.819	0.317	0.941	0.648	0.855	0.715
***SPARCL1* rs1130643 T>C**															
TT	63.995 ± 11.205	24.053 ± 3.184	44.787 ± 16.293	11.053 ± 6.342	6.876 ± 5.000	698.045 ± 318.017	189.567 ± 41.368	151.096 ± 116.538	244.512 ± 91.377	11.974 ± 3.660	30.454 ± 4.208	425.396 ± 126.870	91.998 ± 16.796	15.981 ± 5.513	4.589 ± 1.507
TC	63.682 ± 10.707	25.126 ± 5.970	44.124 ± 12.199	11.931 ± 7.868	6.787 ± 4.795	648.227 ± 342.309	189.793 ± 39.098	149.218 ± 84.572	247.045 ± 68.241	11.714 ± 0.766	31.177 ± 5.348	438.910 ± 144.359	95.591 ± 16.264	17.034 ± 8.814	4.860 ± 1.627
CC	67.500 ± 12.604	22.250 ± 1.921	53.200 ± 13.603	11.269 ± 3.882	4.850 ± 2.472	801.000 ± 320.140	210.143 ± 35.541	174.000 ± 97.913	232.250 ± 82.920	12.075 ± 1.297	29.119 ± 4.688	444.933 ± 176.523	88.947 ± 19.393	17.625 ± 8.259	5.125 ± 1.380
P^a	0.650	**0.025**	0.338	0.527	0.559	0.280	0.419	0.852	0.893	0.795	0.251	0.669	0.186	0.289	0.218

ANOVA, analysis of variance; SD, standard deviation; BMI, body mass index; HDL-C, high-density lipoprotein cholesterol; PLT, platelet; PT, prothrombin time; aPTT, activated partial thromboplastin time; BUN, blood urea nitrogen; *SPARCL1*, secreted protein acidic and rich in cysteine-like 1. [a] *p*-value was calculated using ANOVA. Bold values indicate statistical significance ($p < 0.05$).

Table S8. Stratified analysis using clinical parameters with frequency of *SPARCL1* genotypes

Genotype	Age				Sex				Hypertension				Diabetes mellitus				Hyperlipidemia			
	<63		≥63		Male		Female		No		Yes		No		Yes		No		Yes	
	**AOR(95% CI)***	***P***	**AOR(95% CI)***	***P***	**AOR(95% CI)***	***P***	**AOR(95% CI)***	***P***	**AOR(95% CI)***	***P***	**AOR(95% CI)***	***P***	**AOR(95% CI)***	***P***	**AOR(95% CI)***	***P***	**AOR(95% CI)***	***P***	**AOR(95% CI)***	***P***
***SPARCL1* rs1049539 T>C**																				
TT	1.000 (reference)		1.000 (reference)		1.000 (reference)		1.000 (reference)		1.000 (reference)		1.000 (reference)		1.000 (reference)		1.000 (reference)		1.000 (reference)		1.000 (reference)	
TC	1.052 (0.673 - 1.644)	0.825	1.121 (0.763 - 1.646)	0.561	1.242 (0.802 - 1.921)	0.331	0.914 (0.618 - 1.351)	0.652	0.845 (0.553 - 1.292)	0.438	1.318 (0.885 - 1.961)	0.174	1.153 (0.836 - 1.590)	0.387	0.804 (0.415 - 1.557)	0.518	1.194 (0.855 - 1.668)	0.299	0.766 (0.429 - 1.370)	0.369
CC	0.963 (0.407 - 2.279)	0.931	0.886 (0.436 - 1.801)	0.738	1.338 (0.509 - 3.513)	0.555	0.749 (0.381 - 1.474)	0.403	0.862 (0.387 - 1.922)	0.717	0.995 (0.467 - 2.118)	0.989	0.809 (0.442 - 1.482)	0.492	1.927 (0.376 - 9.865)	0.431	0.992 (0.532 - 1.851)	0.980	0.919 (0.282 - 2.995)	0.888
Dominant (TT vs TC+CC)	1.037 (0.674 - 1.595)	0.869	1.079 (0.747 - 1.559)	0.685	1.252 (0.820 - 1.913)	0.298	0.882 (0.608 - 1.279)	0.508	0.844 (0.562 - 1.266)	0.413	1.269 (0.866 - 1.859)	0.222	1.096 (0.805 - 1.491)	0.561	0.892 (0.466 - 1.705)	0.729	1.156 (0.840 - 1.592)	0.373	0.765 (0.435 - 1.346)	0.353
Recessive (TT+TC vs CC)	0.963 (0.422 - 2.197)	0.928	0.858 (0.431 - 1.708)	0.662	1.231 (0.489 - 3.099)	0.659	0.756 (0.393 - 1.452)	0.400	0.940 (0.433 - 2.042)	0.876	0.885 (0.431 - 1.819)	0.740	0.765 (0.424 - 1.383)	0.376	2.453 (0.513 - 11.727)	0.261	0.897 (0.491 - 1.639)	0.725	0.940 (0.305 - 2.894)	0.914
***SPARCL1* rs7695558 A>G**																				
AA	1.000 (reference)		1.000 (reference)		1.000 (reference)		1.000 (reference)		1.000 (reference)		1.000 (reference)		1.000 (reference)		1.000 (reference)		1.000 (reference)		1.000 (reference)	
AG	0.739 (0.442 - 1.236)	0.249	0.875 (0.550 - 1.392)	0.573	0.688 (0.408 - 1.160)	0.161	0.913 (0.577 - 1.447)	0.699	0.714 (0.427 - 1.192)	0.198	0.896 (0.560 - 1.435)	0.647	0.793 (0.542 - 1.162)	0.234	0.921 (0.414 - 2.048)	0.840	0.778 (0.531 - 1.139)	0.196	0.934 (0.419 - 2.084)	0.868
GG	0.451 (0.100 - 2.039)	0.301	1.357 (0.390 - 4.728)	0.632	0.544 (0.076 - 3.920)	0.546	1.055 (0.368 - 3.023)	0.921	0.649 (0.178 - 2.368)	0.512	1.152 (0.284 - 4.677)	0.843	0.850 (0.320 - 2.256)	0.744	0.866 (0.070 - 10.739)	0.911	0.854 (0.263 - 2.768)	0.792	0.912 (0.216 - 3.856)	0.900
Dominant (AA vs AG+GG)	0.709 (0.432 - 1.165)	0.174	0.908 (0.583 - 1.415)	0.670	0.681 (0.409 - 1.134)	0.140	0.933 (0.605 - 1.440)	0.755	0.705 (0.432 - 1.150)	0.162	0.920 (0.584 - 1.449)	0.719	0.802 (0.557 - 1.154)	0.234	0.910 (0.421 - 1.970)	0.812	0.784 (0.541 - 1.135)	0.197	0.934 (0.453 - 1.925)	0.853
Recessive (AA+AG vs GG)	0.485 (0.109 - 2.166)	0.343	1.357 (0.391 - 4.709)	0.630	0.573 (0.081 - 4.079)	0.578	1.095 (0.384 - 3.122)	0.865	0.691 (0.190 - 2.509)	0.574	1.226 (0.305 - 4.926)	0.774	0.888 (0.335 - 2.353)	0.812	0.820 (0.067 - 10.018)	0.876	0.892 (0.275 - 2.892)	0.849	0.949 (0.226 - 3.983)	0.943
***SPARCL1* rs1049544 G>C**																				
GG	1.000 (reference)		1.000 (reference)		1.000 (reference)		1.000 (reference)		1.000 (reference)		1.000 (reference)		1.000 (reference)		1.000 (reference)		1.000 (reference)		1.000 (reference)	
GC	1.013 (0.637 - 1.610)	0.958	0.868 (0.578 - 1.302)	0.494	0.788 (0.496 - 1.253)	0.314	1.073 (0.712 - 1.617)	0.738	**0.616 (0.395 - 0.962)**	**0.033**	1.314 (0.861 - 2.004)	0.206	0.928 (0.660 - 1.305)	0.668	0.861 (0.429 - 1.726)	0.673	1.191 (0.837 - 1.695)	0.331	**0.433 (0.227 - 0.827)**	**0.011**
CC	0.811 (0.411 - 1.599)	0.545	0.568 (0.322 - 1.003)	0.051	**0.451 (0.228 - 0.892)**	**0.022**	0.820 (0.467 - 1.441)	0.490	**0.510 (0.275 - 0.946)**	**0.033**	0.808 (0.438 - 1.492)	0.495	**0.611 (0.377 - 0.991)**	**0.046**	0.737 (0.274 - 1.983)	0.545	0.671 (0.409 - 1.099)	0.113	0.621 (0.253 - 1.524)	0.298
Dominant (GG vs GC+CC)	0.949 (0.609 - 1.481)	0.819	0.788 (0.537 - 1.156)	0.222	0.697 (0.448 - 1.084)	0.109	1.010 (0.686 - 1.487)	0.961	**0.582 (0.384 - 0.882)**	**0.011**	1.182 (0.791 - 1.765)	0.414	0.836 (0.605 - 1.156)	0.279	0.836 (0.434 - 1.609)	0.592	1.036 (0.742 - 1.445)	0.837	**0.461 (0.249 - 0.853)**	**0.014**
Recessive (GG+GC vs CC)	0.738 (0.402 - 1.354)	0.326	0.633 (0.379 - 1.059)	0.082	**0.518 (0.278 - 0.966)**	**0.039**	0.794 (0.477 - 1.321)	0.375	0.649 (0.372 - 1.132)	0.128	0.701 (0.403 - 1.220)	0.209	**0.626 (0.402 - 0.976)**	**0.039**	0.877 (0.367 - 2.092)	0.767	**0.604 (0.383 - 0.950)**	**0.029**	0.941 (0.420 - 2.107)	0.883
***SPARCL1* rs1130643 T>C**																				
TT	1.000 (reference)		1.000 (reference)		1.000 (reference)		1.000 (reference)		1.000 (reference)		1.000 (reference)		1.000 (reference)		1.000 (reference)		1.000 (reference)		1.000 (reference)	
TC	0.779 (0.458 - 1.325)	0.357	0.793 (0.496 - 1.269)	0.334	0.770 (0.446 - 1.329)	0.347	0.785 (0.494 - 1.246)	0.304	0.664 (0.392 - 1.125)	0.128	0.899 (0.553 - 1.460)	0.666	0.723 (0.488 - 1.072)	0.107	1.124 (0.494 - 2.555)	0.781	0.757 (0.508 - 1.126)	0.169	0.857 (0.400 - 1.837)	0.691
CC	0.651 (0.129 - 3.280)	0.603	0.858 (0.217 - 3.386)	0.827	1.542 (0.086 - 27.549)	0.768	0.757 (0.249 - 2.302)	0.624	0.499 (0.115 - 2.173)	0.355	1.338 (0.250 - 7.168)	0.734	0.658 (0.198 - 2.183)	0.494	1.323 (0.125 - 14.009)	0.816	1.058 (0.308 - 3.637)	0.929	0.417 (0.060 - 2.901)	0.377
Dominant (TT vs TC+CC)	0.772 (0.460 - 1.293)	0.325	0.796 (0.506 - 1.252)	0.324	0.785 (0.457 - 1.348)	0.380	0.780 (0.502 - 1.211)	0.268	0.648 (0.390 - 1.076)	0.094	0.924 (0.577 - 1.481)	0.744	0.718 (0.491 - 1.051)	0.089	1.143 (0.519 - 2.515)	0.741	0.777 (0.528 - 1.143)	0.200	0.787 (0.384 - 1.615)	0.514
Recessive (TT+TC vs CC)	0.745 (0.149 - 3.726)	0.720	0.874 (0.223 - 3.432)	0.847	1.652 (0.097 - 28.164)	0.729	0.799 (0.263 - 2.426)	0.693	0.564 (0.131 - 2.431)	0.443	1.378 (0.258 - 7.352)	0.707	0.711 (0.215 - 2.353)	0.577	1.291 (0.125 - 13.377)	0.831	1.125 (0.327 - 3.876)	0.852	0.423 (0.062 - 2.890)	0.380

**Smoking**				**Total cholesterol**				**Triglyceride**				**HDL-C**				**Homocysteine**			
No		Yes		< 228 mg/dl		≥ 228 mg/dl		< 222 mg/dl		≥ 222 mg/dl		≥ 40(M)/50(F)		< 40(M)/50(F)		< 13.81 μmol/L		≥ 13.81 μmol/L	
**AOR(95% CI)***	***P***	**AOR(95% CI)***	***P***	**AOR(95% CI)***	***P***	**AOR(95% CI)***	***P***	**AOR(95% CI)***	***P***	**AOR(95% CI)***	***P***	**AOR(95% CI)***	***P***	**AOR(95% CI)***	***P***	**AOR(95% CI)***	***P***	**AOR(95% CI)***	***P***
1.000 (reference)		1.000 (reference)		1.000 (reference)		1.000 (reference)		1.000 (reference)		1.000 (reference)		1.000 (reference)		1.000 (reference)		1.000 (reference)		1.000 (reference)	
0.881 (0.612 - 1.269)	0.498	1.473 (0.900 - 2.410)	0.124	1.057 (0.771 - 1.450)	0.730	0.969 (0.444 - 2.117)	0.937	1.107 (0.809 - 1.516)	0.525	0.944 (0.394 - 2.266)	0.898	0.990 (0.578 - 1.697)	0.972	1.355 (0.781 - 2.350)	0.280	0.983 (0.720 - 1.342)	0.914	1.938 (0.832 - 4.514)	0.125
0.808 (0.420 - 1.555)	0.523	1.295 (0.472 - 3.555)	0.616	0.993 (0.553 - 1.783)	0.981	0.436 (0.070 - 2.731)	0.375	0.854 (0.481 - 1.518)	0.591	2.350 (0.177 - 31.140)	0.517	1.342 (0.447 - 4.027)	0.600	1.235 (0.416 - 3.667)	0.704	0.951 (0.530 - 1.706)	0.866	1.239 (0.237 - 6.460)	0.800
0.868 (0.613 - 1.230)	0.426	1.415 (0.880 - 2.273)	0.152	1.047 (0.773 - 1.416)	0.768	0.893 (0.415 - 1.921)	0.772	1.060 (0.785 - 1.431)	0.704	1.018 (0.429 - 2.416)	0.968	1.041 (0.623 - 1.741)	0.878	1.316 (0.775 - 2.235)	0.309	0.974 (0.722 - 1.314)	0.862	1.722 (0.777 - 3.819)	0.181
0.821 (0.436 - 1.545)	0.540	1.064 (0.403 - 2.807)	0.901	0.970 (0.553 - 1.702)	0.915	0.430 (0.067 - 2.769)	0.374	0.807 (0.462 - 1.410)	0.451	2.360 (0.248 - 22.414)	0.455	1.373 (0.472 - 3.998)	0.561	1.015 (0.356 - 2.897)	0.978	0.940 (0.534 - 1.654)	0.829	0.744 (0.158 - 3.508)	0.708
1.000 (reference)		1.000 (reference)		1.000 (reference)		1.000 (reference)		1.000 (reference)		1.000 (reference)		1.000 (reference)		1.000 (reference)		1.000 (reference)		1.000 (reference)	
0.941 (0.609 - 1.453)	0.784	0.612 (0.345 - 1.085)	0.093	0.768 (0.532 - 1.110)	0.160	1.028 (0.349 - 3.028)	0.960	0.832 (0.577 - 1.199)	0.323	0.543 (0.170 - 1.731)	0.301	**0.519 (0.282 - 0.957)**	**0.036**	1.150 (0.585 - 2.262)	0.685	0.820 (0.566 - 1.188)	0.294	0.804 (0.299 - 2.162)	0.666
1.254 (0.419 - 3.754)	0.686	0.351 (0.057 - 2.163)	0.259	0.514 (0.175 - 1.511)	0.226	3.289 (0.307 - 35.208)	0.325	0.980 (0.349 - 2.754)	0.970	0.690 (0.069 - 6.946)	0.753	0.893 (0.220 - 3.617)	0.874	0.373 (0.059 - 2.345)	0.293	0.987 (0.362 - 2.691)	0.979	0.419 (0.050 - 3.525)	0.423
0.973 (0.643 - 1.472)	0.897	0.581 (0.333 - 1.013)	0.056	0.739 (0.518 - 1.055)	0.096	1.284 (0.483 - 3.412)	0.616	0.844 (0.594 - 1.201)	0.347	0.561 (0.192 - 1.641)	0.291	**0.558 (0.314 - 0.993)**	**0.047**	1.048 (0.550 - 1.997)	0.888	0.836 (0.586 - 1.193)	0.324	0.717 (0.286 - 1.798)	0.478
1.306 (0.438 - 3.901)	0.632	0.354 (0.058 - 2.175)	0.262	0.536 (0.183 - 1.572)	0.256	3.247 (0.311 - 33.865)	0.325	0.993 (0.353 - 2.792)	0.989	0.752 (0.082 - 6.913)	0.801	0.961 (0.238 - 3.888)	0.955	0.360 (0.057 - 2.285)	0.279	1.015 (0.372 - 2.767)	0.977	0.448 (0.057 - 3.522)	0.446
1.000 (reference)		1.000 (reference)		1.000 (reference)		1.000 (reference)		1.000 (reference)		1.000 (reference)		1.000 (reference)		1.000 (reference)		1.000 (reference)		1.000 (reference)	
1.093 (0.745 - 1.603)	0.650	0.720 (0.430 - 1.207)	0.213	0.911 (0.653 - 1.272)	0.585	1.054 (0.442 - 2.518)	0.905	0.982 (0.705 - 1.368)	0.913	0.490 (0.187 - 1.284)	0.147	0.671 (0.377 - 1.193)	0.174	1.202 (0.676 - 2.137)	0.532	0.888 (0.638 - 1.235)	0.480	1.188 (0.516 - 2.735)	0.686
0.755 (0.444 - 1.284)	0.300	**0.458 (0.218 - 0.963)**	**0.039**	0.629 (0.393 - 1.007)	0.053	0.390 (0.100 - 1.520)	0.175	0.637 (0.402 - 1.009)	0.055	0.405 (0.080 - 2.041)	0.273	0.879 (0.384 - 2.016)	0.761	0.838 (0.358 - 1.966)	0.685	0.640 (0.404 - 1.014)	0.058	0.951 (0.237 - 3.812)	0.943
0.996 (0.694 - 1.430)	0.985	0.654 (0.399 - 1.071)	0.092	0.830 (0.605 - 1.138)	0.247	0.876 (0.389 - 1.971)	0.748	0.882 (0.645 - 1.206)	0.430	0.494 (0.196 - 1.241)	0.133	0.691 (0.402 - 1.187)	0.180	1.125 (0.650 - 1.947)	0.675	0.821 (0.600 - 1.123)	0.217	1.109 (0.499 - 2.465)	0.799
0.711 (0.440 - 1.148)	0.163	0.561 (0.282 - 1.114)	0.099	0.659 (0.431 - 1.007)	0.054	0.458 (0.142 - 1.480)	0.192	**0.635 (0.417 - 0.966)**	**0.034**	0.841 (0.239 - 2.961)	0.788	0.989 (0.466 - 2.098)	0.977	0.735 (0.333 - 1.625)	0.447	0.684 (0.451 - 1.038)	0.074	0.749 (0.225 - 2.495)	0.638
1.000 (reference)		1.000 (reference)		1.000 (reference)		1.000 (reference)		1.000 (reference)		1.000 (reference)		1.000 (reference)		1.000 (reference)		1.000 (reference)		1.000 (reference)	
0.953 (0.617 - 1.471)	0.827	**0.511 (0.276 - 0.946)**	**0.033**	0.695 (0.474 - 1.019)	0.063	1.583 (0.577 - 4.342)	0.373	0.788 (0.541 - 1.148)	0.214	0.374 (0.111 - 1.264)	0.113	0.630 (0.332 - 1.196)	0.158	0.831 (0.427 - 1.616)	0.585	0.774 (0.530 - 1.130)	0.184	0.833 (0.301 - 2.306)	0.725
0.718 (0.216 - 2.387)	0.588	1.086 (0.136 - 8.671)	0.938	0.485 (0.136 - 1.728)	0.264	1.121 (0.092 - 13.640)	0.929	0.773 (0.237 - 2.523)	0.669	0.735 (0.047 - 11.531)	0.826	0.946 (0.172 - 5.198)	0.949	0.389 (0.033 - 4.613)	0.455	0.710 (0.226 - 2.234)	0.558	0.567 (0.032 - 10.136)	0.700
0.930 (0.612 - 1.412)	0.733	**0.539 (0.297 - 0.980)**	**0.043**	**0.678 (0.468 - 0.984)**	**0.041**	1.505 (0.581 - 3.898)	0.400	0.787 (0.546 - 1.133)	0.197	0.413 (0.132 - 1.292)	0.128	0.655 (0.355 - 1.208)	0.175	0.797 (0.416 - 1.527)	0.494	0.770 (0.534 - 1.110)	0.161	0.791 (0.298 - 2.098)	0.638
0.746 (0.225 - 2.469)	0.631	1.243 (0.156 - 9.884)	0.837	0.542 (0.153 - 1.922)	0.343	0.990 (0.086 - 11.381)	0.993	0.809 (0.247 - 2.649)	0.727	0.797 (0.051 - 12.367)	0.871	1.002 (0.180 - 5.585)	0.998	0.381 (0.033 - 4.480)	0.443	0.762 (0.242 - 2.400)	0.643	0.544 (0.031 - 9.565)	0.677

Vitamin B12				Folate				Platelet				PT†				aPTT†			
> 426 pg/mL		≤ 426 pg/mL		> 3.52 nmol/L		≤ 3.52 nmol/L		< 306 $10^3/\mu\ell$		≥306 $10^3/\mu\ell$		> 11.00 sec		≤ 11.00 sec		> 26.40 sec		≤ 26.40 sec	
**AOR(95% CI)***	***P***	**AOR(95% CI)***	***P***	**AOR(95% CI)***	***P***	**AOR(95% CI)***	***P***	**AOR(95% CI)***	***P***	**AOR(95% CI)***	***P***	**AOR(95% CI)***	***P***	**AOR(95% CI)***	***P***	**AOR(95% CI)***	***P***	**AOR(95% CI)***	***P***
1.000 (reference)		1.000 (reference)		1.000 (reference)		1.000 (reference)		1.000 (reference)		1.000 (reference)		1.000 (reference)		1.000 (reference)		1.000 (reference)		1.000 (reference)	
1.124 (0.819 - 1.543)	0.470	0.715 (0.318 - 1.607)	0.417	1.010 (0.736 - 1.384)	0.953	1.578 (0.621 - 4.015)	0.338	1.070 (0.780 - 1.467)	0.677	1.181 (0.549 - 2.543)	0.671	1.037 (0.732 - 1.469)	0.837	1.226 (0.527 - 2.853)	0.637	1.014 (0.716 - 1.437)	0.937	1.091 (0.441 - 2.702)	0.850
0.875 (0.489 - 1.565)	0.653	0.829 (0.111 - 6.210)	0.855	0.993 (0.558 - 1.764)	0.980	0.682 (0.056 - 8.284)	0.764	0.893 (0.498 - 1.602)	0.704	1.206 (0.216 - 6.734)	0.831	0.961 (0.499 - 1.850)	0.906	0.449 (0.099 - 2.033)	0.299	0.746 (0.403 - 1.383)	0.352	N/A	0.994
1.081 (0.798 - 1.464)	0.615	0.720 (0.327 - 1.586)	0.415	1.005 (0.743 - 1.358)	0.977	1.495 (0.603 - 3.706)	0.386	1.036 (0.765 - 1.403)	0.819	1.184 (0.563 - 2.492)	0.656	1.025 (0.734 - 1.432)	0.885	1.046 (0.475 - 2.300)	0.912	0.966 (0.693 - 1.348)	0.841	1.198 (0.489 - 2.938)	0.692
0.834 (0.477 - 1.457)	0.523	1.056 (0.150 - 7.446)	0.956	0.977 (0.563 - 1.695)	0.933	0.679 (0.062 - 7.466)	0.752	0.848 (0.481 - 1.493)	0.567	1.237 (0.259 - 5.917)	0.790	0.938 (0.503 - 1.752)	0.842	0.401 (0.094 - 1.705)	0.216	0.752 (0.419 - 1.351)	0.340	N/A	0.994
1.000 (reference)		1.000 (reference)		1.000 (reference)		1.000 (reference)		1.000 (reference)		1.000 (reference)		1.000 (reference)		1.000 (reference)		1.000 (reference)		1.000 (reference)	
0.819 (0.562 - 1.194)	0.299	1.055 (0.410 - 2.717)	0.911	0.732 (0.503 - 1.066)	0.103	1.290 (0.418 - 3.980)	0.658	0.767 (0.530 - 1.111)	0.161	1.196 (0.449 - 3.188)	0.720	0.886 (0.593 - 1.323)	0.553	**0.300 (0.109 - 0.820)**	**0.019**	0.746 (0.501 - 1.111)	0.149	1.001 (0.341 - 2.943)	0.998
0.860 (0.316 - 2.341)	0.768	0.682 (0.074 - 6.324)	0.736	0.683 (0.254 - 1.833)	0.449	N/A	N/A	0.682 (0.242 - 1.924)	0.470	1.540 (0.226 - 10.474)	0.659	0.769 (0.262 - 2.256)	0.632	1.057 (0.078 - 14.288)	0.967	0.497 (0.159 - 1.555)	0.230	N/A	0.995
0.823 (0.574 - 1.180)	0.289	1.022 (0.418 - 2.496)	0.963	0.727 (0.508 - 1.042)	0.082	1.565 (0.514 - 4.761)	0.430	0.760 (0.531 - 1.086)	0.132	1.250 (0.510 - 3.062)	0.626	0.871 (0.593 - 1.280)	0.483	**0.345 (0.133 - 0.898)**	**0.029**	0.718 (0.489 - 1.054)	0.091	1.375 (0.499 - 3.786)	0.538
0.895 (0.329 - 2.432)	0.827	0.780 (0.087 - 6.950)	0.823	0.726 (0.270 - 1.949)	0.525	N/A	N/A	0.723 (0.258 - 2.024)	0.537	1.733 (0.251 - 11.939)	0.577	0.770 (0.263 - 2.256)	0.634	1.319 (0.105 - 16.578)	0.830	0.514 (0.164 - 1.613)	0.254	N/A	0.995
1.000 (reference)		1.000 (reference)		1.000 (reference)		1.000 (reference)		1.000 (reference)		1.000 (reference)		1.000 (reference)		1.000 (reference)		1.000 (reference)		1.000 (reference)	
0.837 (0.598 - 1.171)	0.298	1.801 (0.772 - 4.201)	0.173	0.894 (0.640 - 1.250)	0.513	1.296 (0.520 - 3.230)	0.578	0.841 (0.601 - 1.178)	0.314	1.381 (0.629 - 3.035)	0.421	1.049 (0.723 - 1.522)	0.802	0.685 (0.285 - 1.643)	0.397	0.926 (0.638 - 1.343)	0.684	1.032 (0.426 - 2.501)	0.945
**0.626 (0.394 - 0.995)**	**0.048**	0.640 (0.173 - 2.371)	0.504	0.656 (0.412 - 1.045)	0.076	1.240 (0.204 - 7.521)	0.815	**0.600 (0.375 - 0.961)**	**0.033**	0.847 (0.252 - 2.838)	0.787	0.789 (0.473 - 1.315)	0.362	0.271 (0.066 - 1.115)	0.070	0.621 (0.375 - 1.031)	0.066	1.140 (0.250 - 5.195)	0.866
0.777 (0.566 - 1.068)	0.120	1.451 (0.653 - 3.224)	0.361	0.835 (0.609 - 1.146)	0.265	1.255 (0.519 - 3.033)	0.614	0.775 (0.563 - 1.067)	0.119	1.250 (0.593 - 2.632)	0.557	0.970 (0.683 - 1.378)	0.866	0.569 (0.246 - 1.315)	0.187	0.837 (0.589 - 1.189)	0.320	1.079 (0.451 - 2.583)	0.864
0.683 (0.447 - 1.042)	0.077	0.542 (0.174 - 1.683)	0.289	0.712 (0.470 - 1.079)	0.109	0.929 (0.176 - 4.917)	0.931	0.661 (0.434 - 1.009)	0.055	0.683 (0.230 - 2.031)	0.493	0.750 (0.473 - 1.190)	0.222	0.375 (0.114 - 1.235)	0.107	N/A	0.057	1.229 (0.295 - 5.120)	0.777
1.000 (reference)		1.000 (reference)		1.000 (reference)		1.000 (reference)		1.000 (reference)		1.000 (reference)		1.000 (reference)		1.000 (reference)		1.000 (reference)		1.000 (reference)	
0.720 (0.487 - 1.066)	0.101	1.570 (0.604 - 4.079)	0.354	0.710 (0.483 - 1.043)	0.081	1.829 (0.537 - 6.227)	0.334	0.760 (0.518 - 1.116)	0.162	0.964 (0.378 - 2.459)	0.939	0.868 (0.572 - 1.316)	0.505	0.383 (0.144 - 1.015)	0.054	0.698 (0.464 - 1.050)	0.084	1.233 (0.421 - 3.611)	0.702
0.798 (0.265 - 2.401)	0.688	N/A	N/A	0.588 (0.179 - 1.927)	0.380	N/A	N/A	0.903 (0.286 - 2.853)	0.861	0.407 (0.031 - 5.268)	0.491	0.749 (0.226 - 2.486)	0.637	0.763 (0.039 - 14.783)	0.858	0.462 (0.133 - 1.604)	0.224	N/A	0.994
0.728 (0.499 - 1.060)	0.098	1.438 (0.567 - 3.650)	0.445	0.700 (0.483 - 1.016)	0.060	1.984 (0.589 - 6.682)	0.269	0.772 (0.533 - 1.119)	0.172	0.867 (0.356 - 2.116)	0.755	0.857 (0.573 - 1.281)	0.451	0.388 (0.150 - 1.002)	0.050	0.676 (0.455 - 1.004)	0.052	1.463 (0.515 - 4.155)	0.475
0.853 (0.284 - 2.561)	0.777	N/A	N/A	0.636 (0.194 - 2.087)	0.455	N/A	N/A	0.970 (0.308 - 3.054)	0.959	0.423 (0.032 - 5.635)	0.515	0.779 (0.235 - 2.586)	0.684	0.706 (0.039 - 12.731)	0.813	0.498 (0.143 - 1.738)	0.274	N/A	0.994

Fibrinogen ‡				BUN				Uric acid				D-dimer			
< 542 mg/dl		≥ 542 mg/dl		< 20.7 mg/dl		≥ 20.7 mg/dl		< 6.1 mg/dl		≥ 6.1 mg/dl		< 1168.13 ng/mL		≥ 1168.13 ng/mL	
**AOR(95% CI)***	***P***	**AOR(95% CI)***	***P***	**AOR(95% CI)***	***P***	**AOR(95% CI)***	***P***	**AOR(95% CI)***	***P***	**AOR(95% CI)***	***P***	**AOR(95% CI)***	***P***	**AOR(95% CI)***	***P***
1.000 (reference)		1.000 (reference)		1.000 (reference)		1.000 (reference)		1.000 (reference)		1.000 (reference)		1.000 (reference)		1.000 (reference)	
1.420 (0.909 - 2.219)	0.124	0.559 (0.167 - 1.877)	0.347	1.043 (0.762 - 1.427)	0.794	1.051 (0.464 - 2.382)	0.906	1.024 (0.745 - 1.407)	0.886	1.100 (0.480 - 2.520)	0.822	1.505 (0.931 - 2.430)	0.095	0.423 (0.119 - 1.500)	0.183
0.977 (0.430 - 2.217)	0.955	N/A	0.995	0.976 (0.539 - 1.768)	0.937	0.448 (0.086 - 2.328)	0.339	0.862 (0.478 - 1.554)	0.621	1.022 (0.193 - 5.420)	0.980	0.995 (0.416 - 2.383)	0.991	1.777 (0.043 - 73.478)	0.762
1.327 (0.868 - 2.027)	0.191	0.668 (0.201 - 2.219)	0.510	1.029 (0.761 - 1.391)	0.854	0.911 (0.421 - 1.971)	0.813	1.002 (0.738 - 1.358)	0.992	1.097 (0.497 - 2.420)	0.818	1.407 (0.893 - 2.217)	0.141	0.404 (0.116 - 1.405)	0.154
0.832 (0.377 - 1.834)	0.647	N/A	0.995	0.946 (0.533 - 1.680)	0.850	0.390 (0.081 - 1.886)	0.242	0.861 (0.485 - 1.529)	0.610	0.910 (0.186 - 4.449)	0.907	0.825 (0.354 - 1.921)	0.655	0.494 (0.032 - 7.657)	0.614
1.000 (reference)		1.000 (reference)		1.000 (reference)		1.000 (reference)		1.000 (reference)		1.000 (reference)		1.000 (reference)			
**0.580 (0.357 - 0.945)**	**0.029**	1.316 (0.244 - 7.084)	0.749	0.818 (0.563 - 1.190)	0.294	0.709 (0.270 - 1.865)	0.486	0.775 (0.533 - 1.128)	0.184	0.955 (0.356 - 2.562)	0.927	**0.510 (0.301 - 0.864)**	**0.012**	3.327 (0.261 - 42.385)	0.355
0.649 (0.162 - 2.591)	0.540	N/A	0.995	0.744 (0.286 - 1.935)	0.544	N/A	0.995	0.915 (0.336 - 2.492)	0.862	1.888 (0.126 - 28.351)	0.646	1.013 (0.183 - 5.597)	0.989	N/A	0.996
**0.586 (0.365 - 0.939)**	**0.026**	1.588 (0.308 - 8.179)	0.580	0.810 (0.567 - 1.157)	0.246	0.789 (0.306 - 2.036)	0.624	0.788 (0.550 - 1.128)	0.192	1.010 (0.390 - 2.616)	0.984	**0.539 (0.323 - 0.899)**	**0.018**	3.539 (0.287 - 43.633)	0.324
0.695 (0.173 - 2.790)	0.607	N/A	0.995	0.769 (0.296 - 1.995)	0.589	N/A	0.995	0.941 (0.347 - 2.553)	0.905	1.809 (0.123 - 26.540)	0.665	1.148 (0.212 - 6.210)	0.872	N/A	0.996
1.000 (reference)		1.000 (reference)		1.000 (reference)		1.000 (reference)		1.000 (reference)		1.000 (reference)		1.000 (reference)			
0.752 (0.466 - 1.212)	0.241	0.628 (0.176 - 2.239)	0.473	0.835 (0.597 - 1.168)	0.292	1.385 (0.601 - 3.191)	0.445	0.915 (0.653 - 1.283)	0.607	0.859 (0.371 - 1.989)	0.723	0.906 (0.548 - 1.497)	0.699	0.665 (0.170 - 2.594)	0.557
0.759 (0.380 - 1.512)	0.432	N/A	0.996	**0.572 (0.359 - 0.911)**	**0.019**	1.269 (0.370 - 4.345)	0.705	**0.610 (0.385 - 0.966)**	**0.035**	0.633 (0.142 - 2.825)	0.549	0.907 (0.434 - 1.899)	0.796	1.672 (0.151 - 18.461)	0.675
0.759 (0.482 - 1.195)	0.234	0.781 (0.221 - 2.758)	0.701	0.761 (0.553 - 1.046)	0.093	1.321 (0.610 - 2.859)	0.480	0.828 (0.602 - 1.139)	0.246	0.835 (0.373 - 1.869)	0.662	0.901 (0.558 - 1.455)	0.670	0.673 (0.181 - 2.495)	0.553
0.829 (0.440 - 1.563)	0.562	N/A	0.993	**0.632 (0.414 - 0.964)**	**0.033**	1.048 (0.346 - 3.174)	0.935	**0.643 (0.424 - 0.975)**	**0.037**	0.649 (0.180 - 2.340)	0.508	0.937 (0.479 - 1.832)	0.849	0.927 (0.162 - 5.309)	0.932
1.000 (reference)		1.000 (reference)		1.000 (reference)		1.000 (reference)		1.000 (reference)		1.000 (reference)		1.000 (reference)			
**0.577 (0.346 - 0.963)**	**0.036**	1.249 (0.229 - 6.800)	0.797	0.739 (0.503 - 1.085)	0.122	1.107 (0.405 - 3.026)	0.842	0.753 (0.511 - 1.108)	0.150	1.007 (0.364 - 2.785)	0.990	0.579 (0.334 - 1.003)	0.051	4.726 (0.399 - 55.950)	0.218
0.721 (0.134 - 3.875)	0.703	N/A	0.994	0.689 (0.218 - 2.172)	0.524	1.448 (0.112 - 18.725)	0.777	0.658 (0.228 - 1.903)	0.440	N/A	0.994	2.124 (0.235 - 19.174)	0.502	N/A	0.996
**0.585 (0.355 - 0.964)**	**0.035**	1.361 (0.252 - 7.339)	0.720	0.734 (0.507 - 1.064)	0.103	1.128 (0.429 - 2.966)	0.808	0.743 (0.512 - 1.077)	0.117	1.117 (0.411 - 3.035)	0.828	0.628 (0.367 - 1.073)	0.089	4.942 (0.426 - 57.323)	0.201
0.792 (0.148 - 4.245)	0.785	N/A	0.995	0.746 (0.237 - 2.348)	0.616	1.256 (0.099 - 15.884)	0.861	0.709 (0.246 - 2.045)	0.525	N/A	0.994	2.314 (0.259 - 20.689)	0.453	N/A	0.996

AOR, adjusted odds ratio; 95% CI, 95% confidence interval; PT, prothrombin time; aPTT, activated partial thromboplastin time; N/A, not applicable; *SPARCL1*, secreted protein acidic and rich in cysteine-like 1.

* The adjusted odds ratio is based on risk factors, such as age, gender, hypertension, diabetes mellitus, hyperlipidemia, and smoking. Bold values indicate statistical significance ($p < 0.05$) for both p-values and AORs.

† Folate 3.54nmol/L, PT 11.00 sec, aPTT 26.30 sec, and vitamin B12 425 pg/mL were lower 15% cut-off each level in ischemic stroke patients and controls.

‡ Homocysteine 13.7 umol/L, platelet 305 103/$\mu\ell$, fibrinogen 537 mg/dl, total cholesterol 229 mg/dl, triglyceride 221 mg/dl, d-dimer 1264.48 ng/mL, BUN 20.7 mg/dl, and uric acid 6.1 mg/dl were upper 15% cut-off each level in ischemic stroke patients and controls.

Table S9. The synergistic effect analysis of the interplay between clinical factors and *SPARCL1* polymorphisms in ischemic stroke prevalence

Characteristics	*SPARCL1* rs1049539 T>C		*SPARCL1* rs1049539 T>C		*SPARCL1* rs7695558 A>G		*SPARCL1* rs7695558 A>G		*SPARCL1* rs1049544 G>C		*SPARCL1* rs1049544 G>C		*SPARCL1* rs1130643 T>C		*SPARCL1* rs1130643 T>C	
	TT		TC+CC		AA		AG+GG		GG		GC+CC		TT		TC+CC	
	AOR (95% CI)*	*P*	AOR (95% CI)*	*P*	AOR (95% CI)*	*P*	AOR (95% CI)*	*P*	AOR (95% CI)*	*P*	AOR (95% CI)*	*P*	AOR (95% CI)*	*P*	AOR (95% CI)*	*P*
Age																
<63	1.000 (reference)		1.037 (0.674 - 1.595)	0.869	1.000 (reference)		0.699 (0.425 - 1.149)	0.158	1.000 (reference)		0.949 (0.609 - 1.481)	0.819	1.000 (reference)		0.772 (0.460 - 1.293)	0.325
≥63	1.077 (0.710 - 1.634)	0.728	1.205 (0.801 - 1.812)	0.371	1.103 (0.795 - 1.532)	0.556	1.018 (0.630 - 1.644)	0.942	1.526 (0.934 - 2.491)	0.091	1.004 (0.651 - 1.550)	0.984	1.155 (0.836 - 1.597)	0.382	0.959 (0.589 - 1.561)	0.867
Sex																
Male	1.000 (reference)		1.252 (0.820 - 1.913)	0.298	1.000 (reference)		0.681 (0.409 - 1.134)	0.140	1.000 (reference)		0.697 (0.448 - 1.084)	0.109	1.000 (reference)		0.785 (0.457 - 1.348)	0.380
Female	0.979 (0.613 - 1.565)	0.929	0.970 (0.592 - 1.588)	0.902	0.939 (0.636 - 1.388)	0.753	1.070 (0.633 - 1.807)	0.802	0.752 (0.423 - 1.336)	0.331	0.722 (0.438 - 1.191)	0.203	1.007 (0.685 - 1.480)	0.972	0.973 (0.570 - 1.660)	0.920
Hypertension																
No	1.000 (reference)		0.844 (0.562 - 1.266)	0.413	1.000 (reference)		0.705 (0.432 - 1.150)	0.162	1.000 (reference)		**0.582 (0.384 - 0.882)**	**0.011**	1.000 (reference)		0.648 (0.390 - 1.076)	0.094
Yes	**1.890 (1.268 - 2.815)**	**0.002**	**2.477 (1.675 - 3.663)**	**<0.001**	**2.297 (1.661 - 3.174)**	**<0.001**	**2.076 (1.305 - 3.305)**	**0.002**	1.565 (0.973 - 2.517)	0.065	**1.968 (1.311 - 2.955)**	**0.001**	**2.254 (1.639 - 3.099)**	**<0.001**	**2.010 (1.234 - 3.275)**	**0.005**
Diabetes mellitus																
No	1.000 (reference)		1.096 (0.805 - 1.491)	0.561	1.000 (reference)		0.802 (0.557 - 1.154)	0.234	1.000 (reference)		0.836 (0.605 - 1.156)	0.279	1.000 (reference)		0.718 (0.491 - 1.051)	0.089
Yes	**2.259 (1.377 - 3.706)**	**0.001**	**1.932 (1.154 - 3.233)**	**0.012**	**1.933 (1.291 - 2.894)**	**0.001**	1.740 (0.850 - 3.561)	0.130	**2.125 (1.177 - 3.836)**	**0.012**	**1.744 (1.071 - 2.840)**	**0.025**	**1.806 (1.210 - 2.696)**	**0.004**	2.043 (0.984 - 4.240)	0.055
Hyperlipidemia																
No	1.000 (reference)		1.156 (0.840 - 1.592)	0.373	1.000 (reference)		0.784 (0.541 - 1.135)	0.197	1.000 (reference)		1.036 (0.742 - 1.445)	0.837	1.000 (reference)		0.777 (0.528 - 1.143)	0.200
Yes	**1.619 (1.018 - 2.575)**	**0.042**	1.290 (0.829 - 2.007)	0.259	1.291 (0.900 - 1.851)	0.165	1.242 (0.639 - 2.415)	0.523	**2.209 (1.275 - 3.826)**	**0.005**	1.091 (0.699 - 1.703)	0.701	1.339 (0.936 - 1.917)	0.110	1.077 (0.561 - 2.069)	0.823
Smoking																
No	1.000 (reference)		0.868 (0.613 - 1.230)	0.426	1.000 (reference)		0.973 (0.643 - 1.472)	0.897	1.000 (reference)		0.996 (0.694 - 1.430)	0.985	1.000 (reference)		0.930 (0.612 - 1.412)	0.733
Yes	1.115 (0.696 - 1.787)	0.650	1.667 (1.009 - 2.753)	0.046	**1.625 (1.100 - 2.402)**	**0.015**	1.152 (0.656 - 2.022)	0.623	1.603 (0.897 - 2.866)	0.111	1.091 (0.663 - 1.794)	0.732	**1.675 (1.141 - 2.458)**	**0.008**	1.072 (0.584 - 1.965)	0.824
Total cholesterol																
< 228 mg/dl	1.000 (reference)		1.047 (0.773 - 1.416)	0.768	1.000 (reference)		0.739 (0.518 - 1.055)	0.096	1.000 (reference)		0.830 (0.605 - 1.138)	0.247	1.000 (reference)		**0.678 (0.468 - 0.984)**	**0.041**
≥ 228 mg/dl	0.648 (0.342 - 1.227)	0.183	0.639 (0.325 - 1.254)	0.193	0.622 (0.377 - 1.024)	0.062	1.056 (0.443 - 2.520)	0.902	0.490 (0.223 - 1.077)	0.076	0.583 (0.309 - 1.096)	0.094	**0.565 (0.340 - 0.938)**	**0.027**	1.060 (0.460 - 2.442)	0.891
Triglyceride																
< 222 mg/dl	1.000 (reference)		1.060 (0.785 - 1.431)	0.704	1.000 (reference)		0.844 (0.594 - 1.201)	0.347	1.000 (reference)		0.882 (0.645 - 1.206)	0.430	1.000 (reference)		0.787 (0.546 - 1.133)	0.197
≥ 222 mg/dl	0.486 (0.234 - 1.013)	0.054	0.546 (0.286 - 1.046)	0.068	0.646 (0.377 - 1.108)	0.112	0.517 (0.211 - 1.267)	0.149	0.535 (0.239 - 1.200)	0.129	**0.384 (0.191 - 0.772)**	**0.007**	0.642 (0.377 - 1.091)	0.101	0.480 (0.184 - 1.250)	0.133
HDL-c																
≥ 40(M)/50(F)	1.000 (reference)		1.041 (0.623 - 1.741)	0.878	1.000 (reference)		**0.558 (0.314 - 0.993)**	**0.047**	1.000 (reference)		0.691 (0.402 - 1.187)	0.180	1.000 (reference)		0.655 (0.355 - 1.208)	0.175
< 40(M)/50(F)	1.359 (0.809 - 2.282)	0.247	**1.830 (1.084 - 3.090)**	**0.024**	1.380 (0.898 - 2.121)	0.142	1.437 (0.740 - 2.788)	0.284	1.263 (0.687 - 2.321)	0.453	1.415 (0.808 - 2.476)	0.225	1.512 (0.992 - 2.307)	0.055	1.233 (0.635 - 2.394)	0.536
Homocysteine																
< 13.81 μmol/L	1.000 (reference)		0.974 (0.722 - 1.314)	0.862	1.000 (reference)		0.836 (0.586 - 1.193)	0.324	1.000 (reference)		0.821 (0.600 - 1.123)	0.217	1.000 (reference)		0.770 (0.534 - 1.110)	0.161

≥ 13.81 μmol/L	1.141 (0.635 - 2.050)	0.658	**2.036 (1.127 - 3.675)**	**0.018**	1.499 (0.930 - 2.417)	0.097	1.121 (0.515 - 2.438)	0.774	1.204 (0.620 - 2.337)	0.584	1.391 (0.793 - 2.441)	0.250	1.446 (0.909 - 2.302)	0.120	1.126 (0.481 - 2.636)	0.784
Vitamin B12																
> 426 pg/mL	1.000 (reference)		1.081 (0.798 - 1.464)	0.615	1.000 (reference)		0.823 (0.574 - 1.180)	0.289	1.000 (reference)		0.777 (0.566 - 1.068)	0.120	1.000 (reference)		0.728 (0.499 - 1.060)	0.098
≤ 426 pg/mL	1.713 (0.941 - 3.119)	0.078	1.278 (0.726 - 2.252)	0.395	1.383 (0.859 - 2.227)	0.182	1.383 (0.642 - 2.978)	0.407	0.978 (0.499 - 1.914)	0.947	1.458 (0.841 - 2.526)	0.179	1.288 (0.803 - 2.067)	0.294	1.600 (0.730 - 3.505)	0.240
Folate																
> 3.52 nmol/L	1.000 (reference)		1.005 (0.743 - 1.358)	0.977	1.000 (reference)		0.727 (0.508 - 1.042)	0.082	1.000 (reference)		0.835 (0.609 - 1.146)	0.265	1.000 (reference)		0.700 (0.483 - 1.016)	0.060
≤ 3.52 nmol/L	**3.167 (1.694 - 5.920)**	**0.0003**	**5.084 (2.421 - 10.675)**	**<0.0001**	**2.623 (1.547 - 4.445)**	**0.0003**	**5.278 (1.941 - 14.352)**	**0.001**	**2.583 (1.265 - 5.276)**	**0.009**	**3.414 (1.741 - 6.694)**	**0.0004**	**2.748 (1.641 - 4.601)**	**0.0001**	**5.280 (1.744 - 15.989)**	**0.003**
Platelet																
< 306 $10^3/\mu\ell$	1.000 (reference)		1.036 (0.765 - 1.403)	0.819	1.000 (reference)		0.760 (0.531 - 1.086)	0.132	1.000 (reference)		0.775 (0.563 - 1.067)	0.119	1.000 (reference)		0.772 (0.533 - 1.119)	0.172
≥306 $10^3/\mu\ell$	0.882 (0.522 - 1.490)	0.639	0.860 (0.481 - 1.537)	0.610	0.784 (0.505 - 1.217)	0.278	1.002 (0.449 - 2.236)	0.995	0.693 (0.374 - 1.286)	0.245	0.828 (0.478 - 1.434)	0.500	0.852 (0.549 - 1.322)	0.475	0.739 (0.335 - 1.632)	0.455
PT																
> 11.00 sec	1.000 (reference)		1.025 (0.734 - 1.432)	0.885	1.000 (reference)		0.871 (0.593 - 1.280)	0.483	1.000 (reference)		0.970 (0.683 - 1.378)	0.866	1.000 (reference)		0.857 (0.573 - 1.281)	0.451
≤ 11.00 sec	1.206 (0.678 - 2.144)	0.524	1.237 (0.672 - 2.279)	0.495	1.384 (0.856 - 2.237)	0.185	0.510 (0.221 - 1.179)	0.115	1.615 (0.787 - 3.318)	0.192	0.941 (0.541 - 1.638)	0.830	1.376 (0.851 - 2.223)	0.193	0.532 (0.236 - 1.201)	0.129
aPTT																
> 26.40 sec	1.000 (reference)		0.966 (0.693 - 1.348)	0.841	1.000 (reference)		0.718 (0.489 - 1.054)	0.091	1.000 (reference)		0.837 (0.589 - 1.189)	0.320	1.000 (reference)		0.676 (0.455 - 1.004)	0.052
≤ 26.40 sec	0.788 (0.442 - 1.404)	0.418	0.893 (0.455 - 1.752)	0.742	0.784 (0.483 - 1.272)	0.324	0.962 (0.407 - 2.277)	0.931	0.766 (0.383 - 1.530)	0.450	0.781 (0.431 - 1.417)	0.417	0.770 (0.478 - 1.241)	0.283	0.948 (0.385 - 2.338)	0.908
Fibrinogen																
< 542 mg/dl	1.000 (reference)		1.315 (0.861 - 2.010)	0.205	1.000 (reference)		**0.599 (0.373 - 0.962)**	**0.034**	1.000 (reference)		0.759 (0.482 - 1.195)	0.234	1.000 (reference)		**0.585 (0.355 - 0.964)**	**0.035**
≥ 542 mg/dl	1.865 (0.776 - 4.477)	0.163	1.134 (0.457 - 2.812)	0.786	1.076 (0.543 - 2.132)	0.834	1.569 (0.343 - 7.185)	0.562	1.538 (0.543 - 4.355)	0.417	0.962 (0.414 - 2.239)	0.929	1.148 (0.581 - 2.266)	0.692	1.257 (0.269 - 5.878)	0.771
BUN																
< 20.7 mg/dl	1.000 (reference)		1.029 (0.761 - 1.391)	0.854	1.000 (reference)		0.810 (0.567 - 1.157)	0.246	1.000 (reference)		0.761 (0.553 - 1.046)	0.093	1.000 (reference)		0.734 (0.507 - 1.064)	0.103
≥ 20.7 mg/dl	1.169 (0.665 - 2.052)	0.588	1.296 (0.737 - 2.278)	0.368	1.175 (0.744 - 1.856)	0.490	1.139 (0.509 - 2.551)	0.751	0.874 (0.466 - 1.637)	0.673	1.243 (0.712 - 2.171)	0.445	1.086 (0.690 - 1.708)	0.722	1.355 (0.589 - 3.115)	0.475
Uric acid																
< 6.1 mg/dl	1.000 (reference)		1.002 (0.738 - 1.358)	0.992	1.000 (reference)		0.788 (0.550 - 1.128)	0.192	1.000 (reference)		0.828 (0.602 - 1.139)	0.246	1.000 (reference)		0.743 (0.512 - 1.077)	0.117
≥ 6.1 mg/dl	0.800 (0.440 - 1.454)	0.464	0.903 (0.515 - 1.582)	0.721	0.709 (0.445 - 1.131)	0.149	0.763 (0.347 - 1.677)	0.500	0.775 (0.389 - 1.547)	0.470	0.659 (0.378 - 1.147)	0.140	0.724 (0.457 - 1.147)	0.169	0.797 (0.350 - 1.811)	0.587
D-dimer																
< 1168.13 ng/mL	1.000 (reference)		1.407 (0.893 - 2.217)	0.141	1.000 (reference)		**0.539 (0.323 - 0.899)**	**0.018**	1.000 (reference)		0.901 (0.558 - 1.455)	0.670	1.000 (reference)		0.628 (0.367 - 1.073)	0.089
≥ 1168.13 ng/mL	2.486 (0.959 - 6.445)	0.061	0.917 (0.381 - 2.210)	0.847	1.094 (0.552 - 2.170)	0.797	4.230 (0.503 - 35.602)	0.185	1.895 (0.654 - 5.493)	0.239	1.355 (0.544 - 3.378)	0.514	1.042 (0.527 - 2.057)	0.907	4.760 (0.575 - 39.388)	0.148

AOR, adjusted odds ratio; 95% CI, 95% confidence interval; PT, prothrombin time; aPTT, activated partial thromboplastin time; N/A, not applicable; *SPARCL1*, secreted protein acidic and rich in cysteine-like 1.

* The adjusted odds ratio is based on risk factors, such as age, gender, hypertension, diabetes mellitus, hyperlipidemia, and smoking. Bold values indicate statistical significance ($p < 0.05$) for both p-values and AORs.

† Folate 3.54nmol/L, PT 11.00 sec, aPTT 26.30 sec, and vitamin B12 425 pg/mL were lower 15% cut-off each level in ischemic stroke patients and controls.

‡ Homocysteine 13.7 μmol/L, platelet 305 $10^3/\mu\ell$, fibrinogen 537 mg/dl, total cholesterol 229 mg/dl, triglyceride 221 mg/dl, d-dimer 1264.48 ng/mL, BUN 20.7 mg/dl, and uric acid 6.1 mg/dl were upper 15% cut-off each level in ischemic stroke patients and controls.
